# Comparative Study of Acute Lung Injury in COVID-19 and Non-COVID-19 Patients

**DOI:** 10.3389/fmed.2021.666629

**Published:** 2021-08-16

**Authors:** Jianguo Zhang, Xing Huang, Daoyin Ding, Jinhui Zhang, Liusheng Xu, Zhenkui Hu, Wenrong Xu, Zhimin Tao

**Affiliations:** ^1^Jiangsu Province Key Laboratory of Medical Science and Laboratory Medicine, School of Medicine, Jiangsu University, Zhenjiang, China; ^2^Department of Critical Care Medicine, The Affiliated Hospital, Jiangsu University, Zhenjiang, China; ^3^Department of Urology, Center for Evidence-Based and Translational Medicine, Zhongnan Hospital of Wuhan University, Wuhan, China; ^4^Department of Critical Care Medicine, The First People's Hospital of Jiangxia District, Wuhan, China

**Keywords:** COVID-19, intensive care unit, acute lung injury, acute respiratory distress syndrome, treatment

## Abstract

**Background:** Amid the coronavirus disease 2019 (COVID-19) pandemic, we analyzed clinical characteristics of acute lung injury (ALI) in COVID-19 patients and reported their similarity and dissimilarity to those of non-COVID-19 patients in the intensive care unit (ICU).

**Methods:** We reported on 90 COVID-19 and 130 non-COVID-19 ALI patients in the ICUs of multiple centers. Demographic data, medical histories, laboratory findings, and radiological images were analyzed and compared between the two cohorts and within each cohort between survivors and non-survivors. For ALI survivors, clinical characteristics before and after treatment were also compared.

**Findings:** Aberrations in blood parameters, such as leukocytosis, neutrophilia, and thrombocytopenia, were observed in both cohorts. More characteristic abnormalities, including significantly higher red cell distribution width (RDW), C-reactive proteins, and lactic dehydrogenase (LDH) but lower troponin (TnT) and procalcitonin, were observed in the COVID-19 cohort than in the non-COVID-19 cohort, whereas D-dimer levels showed a similar elevation in both cohorts. The COVID-19 cohort also showed more diversified CT patterns where severe features such as consolidations and crazy paving patterns were more frequently observed. Multivariate analysis indicated that age, fever symptom, prothrombin time, procalcitonin, partial pressure of carbon dioxide, oxygenated hemoglobin, and crazy paving patterns in CT scans were independent risk factors associated with COVID-19.

**Interpretation:** Comparison of ALI characteristics between COVID-19 and non-COVID-19 patients in the ICU setting provided insight into the pathogenesis of ALI induced by different risk factors, suggesting distinct treatment plans.

## Background

Following the novel viral pneumonia that broke out in December 2019, the responsible pathogen was identified as severe acute respiratory syndrome (SARS) coronavirus 2 (SARS-CoV-2), and the illness was later named as coronavirus disease 2019 (COVID-19) (1). Strikingly, as of November 10, 2020, COVID-19 has swept across the world, infecting over 50 million people with a death rate exceeding 2.5% (2). With no valid treatment, the COVID-19 pandemic posed an unprecedented challenge to global public health.

Now, we learn that COVID-19 is far more than a typical pulmonary disease. Nevertheless, the highly infective SARS-CoV-2 is mainly transmitted *via* aerosol (3) with the infection beginning predominantly in the lungs, where acute lung injury (ALI) progressed as the illness worsened (4). ALI can develop into acute respiratory distress syndrome (ARDS) as hypoxemia worsens, leading to a high mortality rate among severe ALI patients (5). Studies in the early period of the COVID-19 breakout identified a 67–85% mortality in patients admitted to intensive care units (ICUs), which was attributed to ARDS (6–8). In contrast, general ARDS mortality in ICU patients was estimated as 35.3% (9). Moreover, ARDS mortality after ICU admission in SARS patients was 52.2% (10).

In this study, we focused on the comparison of ALI/ARDS characteristics between COVID-19 and non-COVID-19 patients in the ICU scenario, looking for insight into the heightened death incidence of COVID-19-induced ALI and propose an efficacious treatment plan.

## Methods

### Study Design

**Figure 1:**
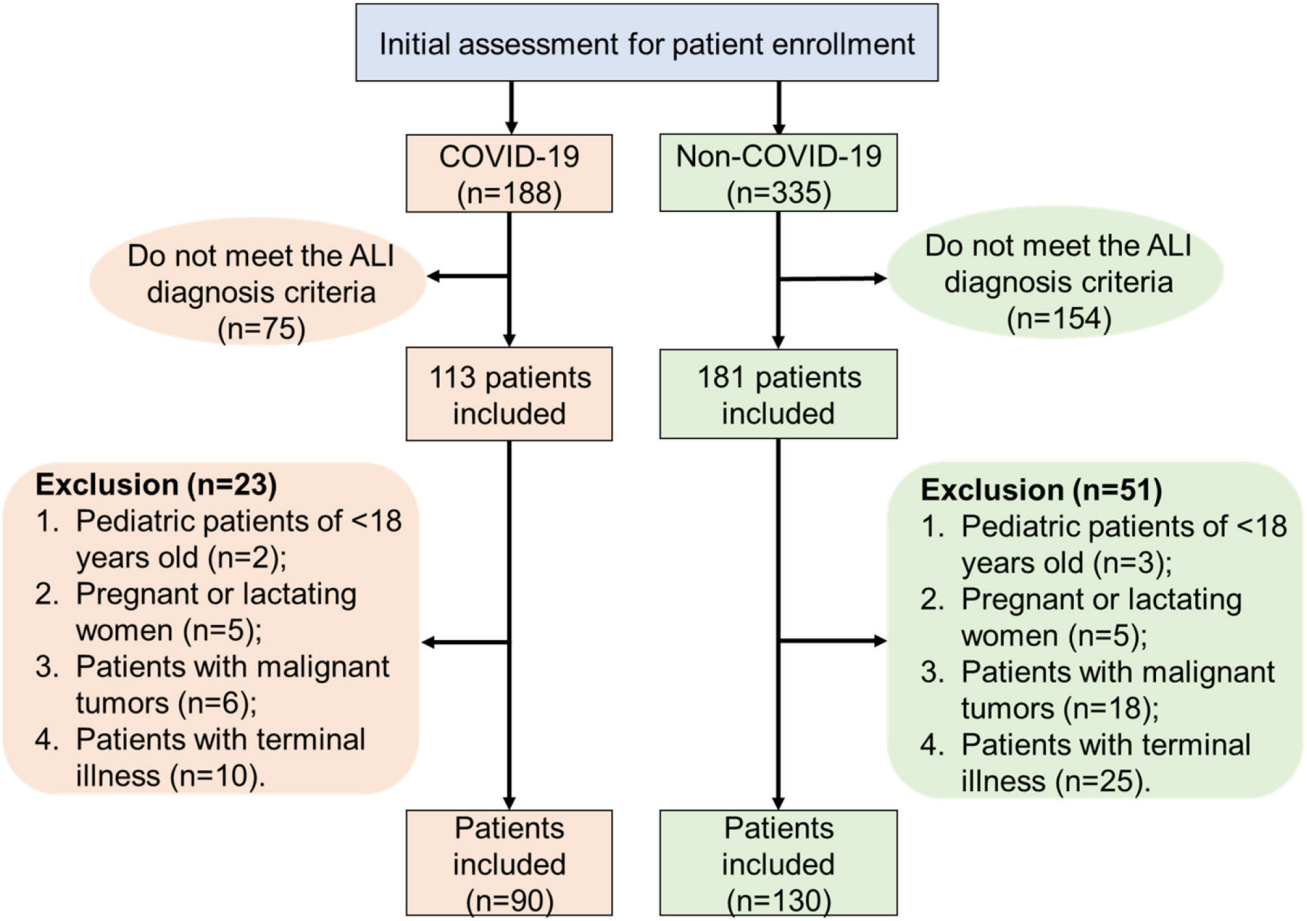
A flowchart that illustrates patient inclusion and exclusion criteria in the patient selection procedure.

In this retrospective study, we reported 90 COVID-19 ALI patients (admitted between January 2020 and April 2020) and 130 non-COVID-19 ALI patients (admitted between January 2017 and October 2019) from different ICUs of multiple centers. For all selected ICU patients, they were diagnosed with ARDS upon ICU admission. ARDS was defined when positive end expiratory pressure (PEEP) or continuous positive airway pressure (CPAP) was >5 cmH_2_O and PaO_2_/FiO_2_ was <300 mmHg, following a classic Berlin Definition (11). Exclusion criteria were as follows: (1) pediatric patients <18 years old; (2) pregnant or lactating women patients; and (3) patients with malignant tumors, immunodeficiency, or terminal illness. A flowchart indicating the inclusion and exclusion criteria of patients is shown in Figure 1. As a result, 130 ALI patients admitted to the ICU in the Affiliated Hospital of Jiangsu University (TAHJU) were selected as the non-COVID-19 cohort. Patient consents were acquired, and the study was approved by the Medical Ethics Committee of TAHJU. In parallel, 90 ALI patients in the COVID-19 cohort were admitted to the First People's Hospital of Jiangxia District (TFPHJD) at Wuhan and Huangshi Central Hospital (HCH) at Huangshi city, both in the Hubei Province of China. ALI/ARDS management followed the published formal guidelines (12–14). Patient information remains anonymous and written consent was waived. The study was approved by the Ethics Commissions of TFPHJD and HCH. Patient data for comparison were gathered before and after stays in ICU. More details of the study design can be found in the Supplementary Material.

### Procedure

Details of patient procedures can be found in the Supplementary Material. In particular, all COVID-19 patients were received at TFPHJD and HCH and diagnosed following the standard procedure, and all COVID-19 ICU patients were admitted following published criteria (15) and treated by following the published guidelines during early outbreak (16). For patients with clinical symptoms, such as fever, cough, and radiological abnormality, throat swabs were gathered for SARS-CoV-2 RNA detection by gene sequencing or by real-time RT-PCR as previously reported (7).

### Statistical Analysis

The categorical variables were described as frequency rates and percentages, and continuous variables were applied to describe the median and interquartile range (IQR) values. All data were collected and compared between the COVID-19 cohort and the non-COVID-19 cohort. Comparison of continuous variables between the two cohorts was analyzed with Mann–Whitney test, and *c*^2^ test was used to compare the categorical variables. These statistical methods followed a published method (17), and methodological details can be found in the Supplementary Material. For key variables with a *p* < 0.05 in the univariate analysis, multivariate logistic regression analysis was performed to explore the independent risk factors associated with either the COVID-19 or the non-COVID-19 cohort. A *p* < 0.05 was considered statistically significant.

## Results

A total of 220 ICU patients were hospitalized, namely, a COVID-19 cohort of 90 and a non-COVID-19 (non-viral) cohort of 130. Median age was 68.0 (IQR: 57.0–76.0); 33.2% were female and 31.8% had a history of smoking (Table 1). The median ICU stay was 13 days, and the eventual mortality rate reached 44.5%. Compared to the non-COVID-19 cohort, the COVID-19 cohort showed younger age, fewer male patients, shorter ICU stays, and higher death rate (*p* < 0.05), although smoking history had a similar effect on both cohorts; 74.5% of patients had comorbidity, with the COVID-19 cohort (65.5%) having a significantly lower proportion of patients with comorbidity than the non-COVID-19 cohort (80.8%). Hypertension, diabetes, bronchitis, and cardiovascular disease were the most common comorbidities. The frequency of each comorbidity showed no significant difference between the two cohorts. Despite different disease pathogeneses, patients in both cohorts experienced similar symptoms, including cough, fever, dyspnea, expectoration, fatigue, and vomiting (Table 1). Notably, the COVID-19 cohort had significantly more patients with fever but fewer showing expectoration. In our previous study, significantly fewer COVID-19 patients experienced expectoration than influenza patients despite sharing flu-like symptoms (17).

**Table 1:** Demographic data, medical history, and clinical symptoms of 220 ALI patients.

	**Total (*n* = 220)**	**COVID-19 (*n* = 90)**	**Non-COVID-19 (*n* = 130)**	***p*-value**
Age	68.0 (57.0–76.0)	60.5 (46.8–71.3)	70.0 (63.8–78.0)	<0.0001
Gender, female *N* (%)	73 (33.2%)	39 (43.3%)	34 (26.2%)	0.008
Smoking history	70 (31.8%)	30 (33.3%)	40 (30.8%)	0.688
ICU stay, day	13.0 (9.0–23.8)	10.0 (6.0–20.3)	15.0 (12.0–27.0)	<0.0001
Mortality, *N* (%)	98 (44.5%)	52 (57.8%)	46 (35.4%)	0.001
**Comorbidity**				
Hypertension	86 (39.1%)	34 (37.8%)	52 (40.0%)	0.740
Diabetes	42 (19.1)	15 (16.7%)	27 (20.8%)	0.447
Bronchitis	31 (14.1%)	9 (10.0%)	22 (16.9%)	0.147
Cardiovascular diseases	24 (10.9%)	6 (6.7%)	18 (13.8%)	0.093
Hepatitis B	9 (4.1%)	3 (3.3%)	6 (4.6%)	0.741
Intracerebral hemorrhage	6 (2.7%)	4 (4.4%)	2 (1.5%)	0.229
Renal dysfunction	6 (2.7%)	2 (2.2%)	4 (3.1%)	1.000
Hypothyroidism	5 (2.3%)	1 (1.1%)	4 (3.1%)	0.651
Gallstone	4 (1.8%)	4 (4.4%)	0 (0)	0.027
Cholecystitis	3 (1.4%)	2 (2.2%)	1 (0.8%)	0.569
Renal calculi	3 (1.4%)	2 (2.2%)	1 (0.8%)	0.569
Gout	2 (0.9%)	2 (2.2%)	0 (0)	0.166
**Symptoms**				
Cough	144 (65.5%)	65 (72.2%)	79 (60.8%)	0.079
Fever	127 (57.7%)	67 (74.4%)	60 (46.2%)	<0.0001
Dyspnea	86 (39.1%)	33 (36.7%)	53 (40.8%)	0.540
Expectoration	77 (35.0%)	22 (24.4%)	55 (42.3%)	0.006
Fatigue	59 (26.8%)	31 (34.4%)	28 (21.5%)	0.034
Vomiting	33 (15.0%)	11 (12.2%)	22 (16.9%)	0.337
Diarrhea	20 (9.1%)	11 (12.2%)	9 (6.9%)	0.179
Chest pain	18 (8.2%)	3 (3.3%)	15 (11.5%)	0.043
Abdominal pain	15 (6.8%)	6 (6.7%)	9 (6.9%)	0.941

Baseline blood characteristics for all patients upon ICU admission are shown in Table 2. Compared to the non-COVID-19 cohort, the COVID-19 cohort showed a higher proportion of patients with leukocytosis or thrombocytopenia, but a lower proportion with neutrophilia or monocytosis. Similarly, both cohorts exhibited an overwhelmingly low red blood cell count and low levels of hemoglobin or hematocrit, indicating serious anemia in ALI patients regardless of pathogenesis. Nevertheless, a notably higher proportion of COVID-19 patients with elevated values of red cell distribution width (RDW) compared with that of non-COVID-19 patients was found, establishing a distinctive feature of COVID-19 infection. This finding was consistent with another report (18). For coagulation factors, abnormally increased prothrombin time, activated partial thromboplastin time, thrombin time, D-dimer level, international normalized ratio, and decreased fibrinogen level were found in a substantial number of ALI patients in both cohorts. Among them, D-dimer elevation has been reported to correlate with the severity of COVID-19 (19, 20). In our study, most ALI patients showed heightened D-dimer levels, but these were indistinguishable between the COVID-19 or non-COVID-19 cohorts. In addition, ALI patients showed reduced protein and ionic concentrations, and augmented levels of many metabolic proteins and enzymatic biomarkers (Table 2), including C-reactive proteins (CRPs), bilirubin, ALT, AST, BUN, LDH, and CPK. Among them, compared to the non-COVID-19 cohort, COVID-19 patients demonstrated much higher levels of CRP and LDH, but a dramatically lower level of TnT and procalcitonin.

**Table 2:** Laboratory testing results of ALI patients in the COVID-19 and non-COVID-19 cohorts.

	**Normal range**	**Total (*n* = 220)**	**COVID-19 (*n* = 90)**	**Non-COVID-19 (*n* = 130)**	***p*-value**
**Blood count panel**					
White blood cells, × 10^9^/L	3.5–9.5	9.2 (5.9–14.7)	7.5 (4.8–14.3)	10.2 (6.9–15.3)	0.003
>9.5		107 (48.6%)	34 (37.8%)	73 (56.2%)	0.007
Neutrophils, × 10^9^/L	1.8–6.3	8.4 (4.4–14.7)	5.9 (3.2–13.1)	9.6 (5.8–15.2)	0.0003
>6.3		136 (61.8%)	42 (46.7%)	94 (72.3%)	0.0001
Lymphocytes, × 10^9^/L	1.1–3.2	0.6 (0.4–1.1)	0.6 (0.4–1.1)	0.6 (0.4–1.1)	0.871
>3.2		7 (3.2%)	5 (5.5%)	2 (1.5%)	0.125
Monocytes, × 10^9^/L	0.1–0.6	0.5 (0.3–0.8)	0.3 (0.2–0.5)	0.6 (0.4–1.0)	<0.0001
>0.6		79 (35.9%)	15 (16.7%)	64 (49.2%)	<0.0001
Eosinophils, × 10^9^/L	0.02–0.52	0.0 (0.0–0.04)	0.0 (0.0–0.03)	0.0 (0.0–0.05)	0.330
>0.52		8(3.6%)	2 (2.2%)	6 (4.6%)	0.476
Basophils, × 10^9^/L	0–0.06	0.0 (0.0–0.02)	0.0 (0.0–0.01)	0.0 (0.0–0.02)	0.537
>0.06		18 (8.2%)	5 (5.6%)	13 (10.0%)	0.319
Red blood cells, × 10^12^/L	4.3–5.8	3.1 (2.7–3.8)	3.4 (2.9–4.0)	3.0 (2.6–3.6)	0.001
<4.3		196 (89.1%)	78 (86.7%)	118 (90.8%)	0.337
Hemoglobin, g/L	130–175	104.0 (84.3–124.0)	105.5 (84.0–122.3)	103.0 (84.8–125.3)	0.761
<130		173 (78.6%)	73 (81.1%)	100 (76.9%)	0.456
Hematocrit, %	40–50	31.4 (26.4–37.9)	31.7 (26.4–36.8)	31.4 (26.5–38.6)	0.358
<40		177 (80.5%)	78 (86.7%)	99 (76.2%)	0.053
MCV, fL	82–100	91.8 (87.2–95.8)	90.0 (86.1–95.8)	92.6 (87.9–95.9)	0.128
<82		15 (6.8%)	7 (7.8%)	8 (6.2%)	0.639
MCH, pg	27–34	30.2 (29.1–31.5)	30.6 (29.6–32.2)	29.8 (28.9–31.3)	0.004
<27		21 (9.5%)	6 (6.7%)	15 (11.5%)	0.227
MCHC, g/L	316–354	326.0 (314.0–338.0)	331.0 (317.0–347.5)	322.0 (310.0–334.0)	0.001
<316		63 (28.6%)	19 (21.1%)	44 (33.8%)	0.040
RDW, %	11.5–17.8	17.0 (13.2–41.5)	42.4 (39.1–47.5)	13.7 (12.5–15.1)	<0.0001
>17.8		102 (46.4%)	89 (98.9%)	13 (10.0%)	<0.0001
Platelets, × 10^9^/L	125–350	158.5 (88.5–242.3)	148.0 (76.0–275.0)	162.0 (99.8–233.3)	0.293
<125		84 (38.2%)	42 (46.7%)	42 (32.3%)	0.031
MPV, fL	7.4–12.5	11.0 (10.0–12.4)	10.8 (9.9–12.6)	11.1 (10.1–12.3)	0.497
>12.5		53 (24.1%)	22 (24.4%)	31 (23.8%)	0.919
PDW, %	9–17	16.4 (15.1–17.0)	16.4 (14.9–17.2)	16.4 (15.2–17.0)	0.948
>17		54 (24.5%)	23 (25.6%)	31 (23.8%)	0.772
**Coagulation panel**					
Prothrombin time, s	9–13	14.1 (12.4–15.7)	14.8 (13.5–17.4)	13.3 (11.6–15.3)	<0.0001
>13		145 (65.9%)	78 (86.7%)	67 (51.5%)	<0.0001
INR	0.8–1.2	1.2 (1.0–1.4)	1.2 (1.1–1.5)	1.2 (1.0–1.4)	0.497
>1.2		104 (47.3%)	42 (46.7%)	62 (47.7%)	0.881
aPPT, s	23.3–32.5	30.7 (26.6–37.0)	31.7 (28.6–37.1)	29.2 (24.5–37.7)	0.021
>32.5		92 (41.8%)	39 (43.3%)	53 (40.8%)	0.705
Thrombin time, s	14–21	18.1 (16.8–20.3)	17.4 (15.9–18.9)	18.9 (17.5–22.0)	<0.0001
>21		50 (22.7%)	10 (11.1%)	40 (30.8%)	0.001
Fibrinogen, g/L	2–4	4.3 (2.7–5.7)	4.8 (3.8–5.8)	3.8 (2.2–5.5)	0.012
<2		28 (12.7%)	5 (5.6%)	23 (17.7%)	0.008
D-dimer, mg/L	<0.55	3.4 (0.9–7.5)	3.6 (0.8–71)	2.8 (1.0–8.5)	0.738
>0.55		180 (81.8%)	71 (78.9%)	109 (83.8%)	0.349
**Metabolic panel**					
C-reactive protein, mg/L	0–10	26.7 (7.8–99.1)	45.8 (14.2–88.0)	18.9 (4.0–120.6)	0.026
>10		160 (72.7%)	78 (86.7%)	82 (63.1%)	0.0001
Total bilirubin, mmol/L	3–22	14.1 (7.9–23.1)	12.9 (7.7–22.9)	14.4 (8.0–23.3)	0.421
>22		58 (26.4%)	24 (26.7%)	34 (26.2%)	0.932
Direct bilirubin, mmol/L	0–5	4.7 (2.9–8.5)	5.3 (2.8–8.6)	4.5 (2.9–8.3)	0.838
>5		102 (46.4%)	46 (51.1%)	56 (43.1%)	0.240
Indirect bilirubin, mmol/L	0–19	11.1 (7.8–19.8)	9.7 (6.6–13.4)	13.2 (8.9–46.2)	<0.0001
>19		58 (26.4%)	5 (5.6%)	53 (40.8%)	<0.0001
ALT, U/L	9–50	41.1 (20.6–71.9)	40.7 (17.1–68.9)	42.5 (22.8–72.3)	0.672
>50		84 (38.2%)	32 (35.6%)	52 (40.0%)	0.505
AST, U/L	15–40	49.8 (29.0–79.4)	46.0 (25.8–81.7)	54.3 (30.2–79.3)	0.686
>40		130 (59.1%)	53 (58.9%)	77 (59.2%)	0.960
ALP, U/L	32–126	94.1 (65.0–146.5)	90.5 (65.0–124.3)	103.0 (63.8–164.3)	0.080
>126		72 (32.7%)	19 (21.1%)	53 (40.8%)	0.002
GGT, U/L	12–73	53.5 (31.0–88.8)	45.8 (29.3–81.3)	61.0 (31.8–91.5)	0.136
>73		78 (35.5%)	28 (31.1%)	50 (38.5%)	0.263
Total protein, g/L	65–85	55.1 (49.1–63.3)	57.5 (50.6–64.6)	54.5 (46.2–62.2)	0.010
<65		171 (77.7%)	68 (75.6%)	103 (79.2%)	0.520
Albumin, g/L	40–55	30.0 (26.3–34.7)	31.9 (28.9–35.9)	28.0 (24.4–32.6)	<0.0001
<40		202 (91.8%)	82 (91.1%)	120 (92.3%)	0.750
Globulin, g/L	20–40	24.5 (20.5–29.2)	22.7 (19.2–28.7)	25.5 (21.9–29.2)	0.028
<20		46 (20.9%)	26 (28.9%)	20 (15.4%)	0.015
BUN, mmol/L	2.86–8.2	8.4 (5.4–13.2)	7.7 (4.8–11.6)	8.9 (6.5–14.7)	0.014
>8.2		115 (52.3%)	40 (44.4%)	75 (57.7%)	0.053
Creatinine, mmol/L	31.7–133	71.3 (55.1–103.7)	69.5 (57.8–109.0)	72.2 (53.6–99.7)	0.676
>133		36 (16.4%)	17 (18.9%)	19 (14.6%)	0.400
Carbon dioxide, mmol/L	20–29	25.0 (19.5–29.3)	22.0 (18.4–27.1)	26.0 (21.5–30.3)	0.006
>29		57 (25.9%)	17 (18.9%)	40 (30.8%)	0.048
Glucose, mmol/L	3.89–6.11	8.4 (6.3–12.2)	8.5 (6.8–12.2)	8.1 (5.8–12.3)	0.316
>6.11		170 (77.3%)	76 (84.4%)	94 (72.3%)	0.035
Potassium, mmol/L	3.5–5.3	3.9 (3.5–4.4)	3.9 (3.5–4.4)	3.9 (3.5–4.4)	0.753
<3.5		50 (22.7%)	22 (24.4%)	28 (21.5%)	0.613
Sodium, mmol/L	137–147	138.0 (134.4–142.1)	139.0 (135.1–143.0)	136.7 (133.6–141.0)	0.029
<137		98 (44.5%)	31 (34.4%)	67 (51.5%)	0.012
Total calcium, mmol/L	2.08–2.6	2.0 (1.9–2.1)	1.9 (1.8–2.1)	2.1 (1.9–2.3)	<0.0001
<2.08		144 (65.5%)	71 (78.9%)	73 (56.2%)	0.001
**Biomarkers**					
LDH, U/L	80–285	282.5 (199.3–420.8)	404 (228.2–619.6)	246.5 (178.0–335.5)	<0.0001
>285		107 (48.6%)	59 (65.6%)	48 (36.9%)	<0.0001
TnT, ng/mL	0–0.4	0.13 (0.03–0.94)	0.04 (0.01–0.20)	0.36 (0.07–1.27)	<0.0001
>0.4		76 (34.5%)	13 (14.4%)	63 (48.5%)	<0.0001
Myoglobin, U/L	25–58	73.8 (25.8–217.0)	64.2 (21.1–225.1)	82.9 (27.4–207.3)	0.474
>58		125 (56.8%)	48 (53.3%)	77 (59.2%)	0.385
CPK, U/L	38–174	126.5 (65.0–328.0)	114.9 (53.0–272.8)	165.5 (71.0–328.0)	0.146
>174		89 (40.5%)	30 (33.3%)	59 (45.4%)	0.073
CK-MB, U/L	0–25	25.4 (15.9–43.2)	21.1 (13.1–32.1)	29.1 (18.2–60.7)	0.003
>25		111 (50.5%)	36 (40.0%)	75 (57.7%)	0.010
Homocysteine, mmol/L	0–15	15.0 (12.8–23.5)	15.1 (12.8–22.7)	15.0 (12.8–23.8)	0.997
>15		108 (49.1%)	46 (51.1%)	62 (47.7%)	0.618
Procalcitonin, ng/mL	<0.1	5.1 (0.6–20.9)	0.4 (0.1–1.6)	18.8 (5.5–25.8)	<0.0001
>0.1		201 (91.4%)	71 (78.9%)	130 (100.0%)	<0.0001

*MCV, mean corpuscular volume; MCH, mean corpuscular hemoglobin; MCHC, mean corpuscular hemoglobin concentration; RDW, red cell distribution width; MPV, mean platelet volume; PDW, platelet distribution width; ALT, alanine aminotransferase; AST, aspartate aminotransferase; ALP, alkaline phosphatase; GGT, γ-glutamyl transferase; BUN, blood urea nitrogen; troponin T, TnT; CK-MB, creatine kinase isoenzyme; LDH, lactic dehydrogenase; CPK, creatine phosphokinase; INR, international normalized ratio; aPTT, activated partial thromboplastin time*.

Next, arterial blood gas profiles were examined for all ICU ALI patients (Table 3). Compared to the non-COVID-19 cohort, the COVID-19 cohort exhibited similar levels of blood parameters such as acidity and base excess but significantly lower levels of actual bicarbonate, partial pressure of carbon dioxide or oxygen, oxygen saturation, and oxygenated hemoglobin. In parallel, CT examination was performed for all patients upon ICU admission and image patterns were compared between the two cohorts (Table 4). The COVID-19 cohort showed infections with substantially expanded lung involvement, with a significantly higher portion of ALI patients with bilateral lung involvement, multilobular lesions (with lobe number = 4, 5), and more lesions in each lobe. Specific CT patterns, such as consolidation and pleural effusion, were found significantly more frequently in the COVID-19 cohort. More characteristically, crazy paving patterns, linear opacity, rounded opacity, halo sign, nodules, tree-in-bud sign, air bronchogram, and interlobular septal thickening were more frequently observed in the COVID-19 cohort than the non-COVID-19 cohort, highlighting explicit CT features caused by SARS-CoV-2 infection.

**Table 3:** Arterial blood gas profiles for ALI patients in the COVID-19 and non-COVID-19 cohorts.

	**Normal range**	**Total (*n* = 220)**	**COVID-19 (*n* = 90)**	**Non-COVID-19 (*n* = 130)**	***p*-value**
**ICU panel**					
pH	7.35–7.45	7.30 (7.25–7.33)	7.31 (7.26–7.34)	7.30 (7.25–7.32)	0.103
<7.35		192 (87.3%)	74 (82.2%)	118 (90.8%)	0.062
>7.45		11 (5.0%)	6 (6.7%)	5 (3.8%)	0.363
Base excess, mmol/L	−3–3	−5.2 (−8.1–1.8)	−5.8 (−9.4–1.5)	−4.5 (−7.3–1.9)	0.068
< -3		128 (58.2%)	58 (64.4%)	70 (53.8%)	0.117
>3		47 (21.4%)	19 (21.1%)	28 (21.5%)	0.939
[${\text{aHCO}}_{3}^{-}$], mmol/L	22–27	20.9 (18.3–25.1)	19.7 (17.9–23.5)	22.6 (19.2–25.8)	0.004
<22		121 (55.0%%)	61 (67.8%)	60 (46.2%)	0.002
>27		40 (18.2%)	14 (15.6%)	26 (20.0%)	0.401
PaO_2_, mmHg	80–100	62.6 (58.0–66.3)	60.7 (56.4–65.2)	62.7 (59.4–67.6)	0.006
<80		206 (93.6%)	90 (100.0%)	116 (89.2%)	0.001
PaCO_2_, mmHg	35–45	49.3 (42.5–55.0)	47.4 (38.9–53.7)	50.5 (44.9–56.6)	0.004
<35		13 (5.9%)	10 (11.1%)	3 (2.3%)	0.009
>45		147 (66.8%)	50 (55.6%)	97 (74.6%)	0.003
SO_2_, %	95–100	92.0 (88.2–94.0)	90.5 (87.0–93.3)	92.0 (90.0–94.0)	0.012
<95		186 (84.5%)	79 (87.8%)	107 (82.3%)	0.270
PaO_2_/FiO_2_	>300	217.3 (197.6–247.3)	218.6 (197.3–253.1)	211.9 (197.6–239.5)	0.367
≤ 300		220 (100.0%)	90 (100.0%)	130 (100.0%)	
aADO_2_, mmHg	0–100	90.4 (78.4–107.4)	92.9 (79.7–108.5)	89.2 (76.8–105.7)	0.269
>100		78 (35.5%)	34 (37.8%)	44 (33.8%)	0.549
HbO_2_,%	90–95	85.5 (80.9–89.3)	81.5 (76.5–85.5)	87.3 (84.8–90.4)	<0.0001
<90		176 (80.0%)	83 (92.2%)	93 (71.5%)	0.0002
MetHb, g/dL	0.2–0.8	0.4 (0.3–0.6)	0.4 (0.3–0.6)	0.4 (0.3–0.5)	0.094
<0.2		9 (4.1%)	3 (3.3%)	6 (4.6%)	0.741
tHb, g/dL	11.5–17.4	9.1 (8.2–9.9)	8.7 (7.5–10.0)	9.2 (8.3–9.8)	0.212
<11.5		208 (94.5%)	81 (90.0%)	127 (97.7%)	0.017

*Actual bicarbonate, [${\text{aHCO}}_{3}^{-}$]; PaCO_2_, the partial pressure of carbon dioxide; PaO_2_, the partial pressure of oxygen; SO_2_, the oxygen saturation; PaO_2_/FiO_2_, the oxygenation index; aADO_2_, alveolar-arterial oxygen pressure; tHb, the total hemoglobin; HbO_2_, the oxygenated hemoglobin; MetHb, methemoglobin*.

**Table 4:** Radiological findings of ALI patients in the COVID-19 and non-COVID-19 cohorts.

	**Total (*n* = 220)**	**COVID-19 (*n* = 90)**	**Non-COVID-19 (*n* = 130)**	***p*-value**
**Lung involvement**				
Unilateral	88 (40.0%)	21 (23.3%)	67 (51.5%)	<0.0001
Bilateral	132 (60.0%)	69 (76.7%)	63 (48.5%)	<0.0001
**Number of lobes with lesions**				
0	0	0	0	
1	42 (19.1%)	9 (10.0%)	33 (25.4%)	0.004
2	55 (25.0%)	11 (12.2%)	44 (20.0%)	0.0003
3	38 (17.3%)	13 (14.4%)	25 (19.2%)	0.356
4	68 (30.9%)	45 (50.0%)	23 (17.7%)	<0.0001
5	17 (7.7%)	12 (13.3%)	5 (3.8%)	0.018
**Location of lesions**				
Left upper lobe	79 (35.9%)	39 (43.3%)	40 (30.8%)	0.056
Left lower lobe	150 (68.2%)	69 (76.7%)	81 (62.3%)	0.025
Right upper lobe	75 (34.1%)	43 (47.8%)	32 (24.6%)	0.0004
Right middle lobe	151 (68.6%)	78 (86.7%)	73 (56.2%)	<0.0001
Right lower lobe	168 (76.4%)	81 (90.0%)	87 (66.9%)	<0.0001
**Predominant distribution**
Central	41 (18.6%)	11 (12.2%)	30 (23.1%)	0.042
Peripheral	93 (42.3%)	38 (42.2%)	55 (42.3%)	0.990
Central + Peripheral	87 (39.5%)	42 (46.7%)	45 (34.6%)	0.072
**Characteristic pattern**				
Ground glass opacity (GGO)	93 (42.3%)	31 (34.4%)	62 (47.7%)	0.051
Consolidation	65 (29.5%)	34 (37.8%)	31 (23.8%)	0.026
GGO + Consolidation	50 (22.7%)	25 (27.8%)	25 (19.2%)	0.137
Crazy paving pattern	36 (16.4%)	32 (35.6%)	4 (3.1%)	<0.0001
Linear opacities	84 (38.2%)	58 (64.4%)	26 (20.0%)	<0.0001
Rounded opacities	49 (22.3%)	45 (50.0%)	4 (3.1%)	<0.0001
Halo sign	28 (12.7%)	25 (27.8%)	3 (2.3%)	<0.0001
Nodules	35 (15.9%)	29 (32.2%)	6 (4.6%)	<0.0001
Tree-in-bud sign	19 (8.6%)	16 (17.8%)	3 (2.3%)	<0.0001
Air bronchogram	44 (20.0%)	31 (34.4%)	13 (10.0%)	<0.0001
Interlobular septal thickening	66 (30.0%)	56 (62.2%)	10 (7.7%)	<0.0001
Bronchiolar wall thickening	42 (19.1%)	34 (37.8%)	8 (61.5%)	<0.0001
Cavitation	11 (5.0%)	7 (7.8%)	4 (3.1%)	0.129
Pleural effusion	53 (24.1%)	29 (32.2%)	24 (18.5%)	0.019
Pericardial effusion	16 (7.3%)	9 (10.0%)	7 (5.4%)	0.195

Variables with a *p* < 0.05 in the previous univariate analysis were put into multivariate logistic regression analysis, and results are shown in Table 5. It can be concluded that age, fever symptom, prothrombin time, procalcitonin, PaCO_2_, HbO_2_, and crazy paving patterns in CT scans are independent risk factors for differentiating COVID-19 ALI patients from non-COVID-19 ALI patients. Compared to the non-COVID-19 cohort, the COVID-19 cohort exhibited more inclination to younger population, experiencing fever, lengthened prothrombin time, and augmented lung involvement and crazy paving patterns in CT features. In addition, the COVID-19 patients also showed higher disposition to demonstrate abnormally lower levels of procalcitonin, PaCO_2_, and HbO_2_.

**Table 5:** Multivariate analysis of independent risk factors for differentiating COVID-19 ALI from non-COVID-19 ALI cases.

**Variables**	**Odds ratio (OR)**	**95% confidence interval (CI)**	***p*-value**
Age	0.947	0.912–0.984	0.005
Gender	1.712	0.451–6.500	0.429
Fever	6.283	1.573–25.090	0.009
White blood cells	0.980	0.892–1.076	0.666
Prothrombin time	1.162	1.051–1.286	0.003
TnT	0.589	0.315–1.104	0.099
CK-MB	1.004	0.996–1.013	0.303
Procalcitonin	0.845	0.785–0.909	<0.001
PaCO_2_	0.842	0.759–0.933	0.001
HbO_2_	0.642	0.533–0.775	<0.001
Lung involvement	3.746	0.846–16.592	0.082
Crazy paving pattern	32.169	4.558–227.056	<0.001

Critically ill ALI patients typically developed hypoxemia, dyspnea, and even respiratory failure requiring invasive or non-invasive oxygen support (Table 6). For the COVID-19 cohort, patients were treated with an array of antiviral drugs, including 16.7% with oseltamivir, 44.4% with arbidol, 53.3% with ribavirin, and 61.1% with α-interferon. They were also given a variety of antibiotics, including 18.9% with sulbactam/cefoperazone sodium, 38.9% with piperacillin/tazobactam sodium, 43.3% with imipenem/cilastatin, and 50.0% with moxifloxacin. For the non-COVID-19 cohort, 50% of patients were given imipenem/cilastatin, 32.3% ceftazidime, 30.0% piperacillin/sulbactam sodium, and 26.2% tigecycline. As a result, mortality was 57.8% in the COVID-19 cohort and 35.4% in the non-COVID-19 cohort.

**Table 6:** Treatment of ALI patients in the COVID-19 and non-COVID-19 cohorts.

	**Total (*n* = 220)**	**COVID-19 (*n* = 90)**	**Non-COVID-19 (*n* = 130)**	***p*-value**
**Oxygen support**				
Invasive	146 (66.4%)	56 (62.2%)	90 (69.2%)	0.279
Non-invasive	74 (33.6%)	34 (37.8%)	40 (30.8%)	
**Antibiotics**				
Sulbactam/cefoperazone sodium	17 (7.7%)	17 (18.9%)	0	
Moxifloxacin	45 (20.5%)	45 (50.0%)	0	
Piperacillin/tazobactam sodium	35 (15.9%)	35 (38.9%)	0	
Imipenem/cilastatin	104 (47.3%)	39 (43.3%)	65 (50.0%)	
Piperacillin/sulbactam sodium	39 (17.7%)	0	39 (30.0%)	
Ceftazidime	42 (19.1%)	0	42 (32.3%)	
Tigecycline	34 (15.5%)	0	34 (26.2%)	
**Antiviral drugs**				
Oseltamivir		15 (16.7%)	0	
Ribavirin		48 (53.3%)	0	
α-interferon		55 (61.1%)	0	
Arbidol		40 (44.4%)	0	
**Sedatives**				
Dexmedetomidine		0	79 (60.8%)	
Midazolam		0	35 (26.9%)	
Propofol		0	42 (32.3%)	

Within the COVID-19 cohort, baseline characteristics and radiological parameters were compared between survivors and non-survivors (Supplementary Tables 1–5). High age was found as a risk factor for mortality, while no substantial difference was found between survivors and non-survivors in their other demographic information, medical history, clinical symptoms, and CT patterns upon ICU admission. Between survivors and non-survivors, blood parameters were found to be similar; however, many arterial blood gas features were significantly different. Compared to survivors, non-survivors exhibited lower pH, PaO_2_, SO_2_, PaO_2_/FiO_2_, aADO_2_, HbO_2_, and tHb, but higher PaCO_2_, portending more severely impaired gas exchange in their virus-infected lungs.

In parallel, within the non-COVID-19 cohort, baseline characteristics and radiological parameters were compared between survivors and non-survivors (Supplementary Tables 6–10). Instead of high age, male gender was found to be a risk factor for mortality, while no substantial difference was found between survivors and non-survivors in their other demographic information, co-existing disease, clinical symptoms, and most CT patterns. Paradoxically, many blood parameters were found to be worse in survivors than in non-survivors upon ICU admission, such as aberrantly higher values of white blood cells, neutrophils, D-dimers, LDH, CRP, and procalcitonin, and lower values of PaO_2_/FiO_2_, aADO_2_, and HbO_2_. This could be associated with various pathogeneses of non-COVID-19 ALI (Supplementary Table 10), including direct and indirect lung infection, mostly triggered by sepsis, and leading to various impacts on the patient after ICU admission.

After different treatment plans were adopted in the two cohorts, all arterial blood gas profiles in ALI survivors recovered well and their laboratory parameters and CT characters were significantly ameliorated upon transfer to non-ICU wards (Supplementary Tables 11–16). However, in the COVID-19 cohort, RDW, D-dimer, CRP, and procalcitonin were similarly abnormal compared to before treatment, showing a slow recovery in those values due to COVID-19 infection despite such ICU patients having been discharged from critical care. In contrast, D-dimer, CRP, and procalcitonin were significantly improved in survivors of the non-COVID-19 cohort after treatment.

## Discussion

As pulmonary injuries (e.g., pneumonia and aspiration) may cause direct damage to alveolar epithelium, extrapulmonary insults (e.g., systemic infection, trauma, or other non-pulmonary acute disease) could pose an indirect threat to the integrity of the capillary endothelium. Such impairment can lead to the production of pro-inflammatory cytokines, induction of cell death and leakage at intercellular junctions in the alveolar capillary membrane, and eventual migration of immune cells from microvessels into the alveolar airspace that initiates diffuse alveolar damage (DAD) (21). In the early stage of DAD, an exudative phase takes place where polymorphonuclear leukocytes (e.g., neutrophils and eosinophils), platelets, and plasma proteins in the alveolar capillary are recruited across the damaged ACM to flood interstitium and airspace, interacting with resident macrophages and forming edema (22). Consisting of cell debris, surfactant, cytokines, and other proteins, edema further promotes the formation of hyaline membrane that deposits along the alveolar walls and becomes characteristic of DAD, radiologically featured as patchy ground glass densities (23, 24). During this phase, the initial inflammation from primary insults are exacerbated, and gaseous exchange is seriously impeded.

Then, a proliferative phase follows as a self-repair mechanism when type II pneumocytes start to proliferate and differentiate into type I pneumocytes, to pump the edema into interstitium for drainage, to reproduce surfactants to lower pulmonary tension, and to summon macrophages to clear cell fragments (25, 26). As a result, the permeability barrier of the ACM may recover with improved oxygenation. Conversely, inability to clear alveolar fluid will lead to hypoxemia and hypercapnic acidosis resulting in acute respiratory failure.

In this study, compared to the COVID-19 cohort, the non-COVID-19 patients exhibited higher age, higher male ratio, longer ICU stay, and lower death rate, suggesting a higher incidence of non-viral ALI associated with older age and male gender, consistent with a previous report (5). While common clinical symptoms may include fever, dry cough, dyspnea, fatigue, and diarrhea for both cohorts, a much higher proportion of COVID-19 patients may experience fever but not expectoration.

ALI in the COVID-19 cohort is induced by SARS-CoV-2 infection, a direct pulmonary injury to the patients. In the non-COVID-19 (non-viral) cohort, due to the diversity of primary disease. ALI may be caused by trauma, surgery (non-thoracic or thoracic), and gastrointestinal bleeding (non-pulmonary sepsis), showing a mixture of extrapulmonary and pulmonary induction of acute injury. For hospitalized ALI patients, leading comorbidities include hypertension, diabetes, bronchitis, and cardiovascular diseases, indicating an elevated instability of ACM in those with compromised immune systems.

Besides commonly observed aberrations in blood parameters due to systemic infection, such as leukocytosis, neutrophilia, and thrombocytopenia, more characteristic abnormalities in COVID-19 ALI patients were noticed when compared to their non-COVID-19 counterparts, including significantly higher RDW, CRP, and LDH but lower TnT and procalcitonin, whereas D-dimer levels showed similar elevation between the two cohorts. Furthermore, debilitated oxygenation in arterial blood was noticed more commonly in the COVID-19 cohort than in the non-COVID-19 cohort. After individual treatment and discharge from ICU, those characteristic abnormalities were ameliorated in the non-COVID-19 cohort and to a much lesser degree in the COVID-19 cohort where the characteristic parameters remained markedly out of the normal range, demonstrating a more sluggish recovery from direct lung infection by SARS-CoV-2.

Thoracic CT scan has been recommended as a diagnostic standard of positive COVID-19 following initial nucleic acid testing of pathogen (15, 27). Both asymptomatic and symptomatic COVID-19 patients demonstrated abnormality in CT images, typically progressing from unilateral or bilateral and multifocal ground glass opacities (GGOs) to intensified consolidation, until formation of reticular pave pattern (28, 29). In our study, COVID-19 patients showed more diversified and complicated CT patterns, with severe features such as consolidations and crazy paving patterns in comparison with non-COVID-19 patients.

Possible correlation between specific genes and the incidence of ALI/ARDS was unclear, except that the angiotensin-converting enzyme 2 (ACE2), actively expressed in alveolar epithelial and endothelial cells, is responsible for adjusting alveolar permeability and repairing lung injury and was also identified as the viral entry receptor for SARS-CoV and SARS-CoV-2 (30, 31). In our study, the COVID-19 cohort showed more severe ALI/ARDS with higher mortality and slower recovery among survivors, possibly because inhaled SARS-CoV-2 directly bound and downregulated ACE2, further weakening the lungs (32, 33). To further differentiate COVID-19 ALI characteristics from non-COVID-19 cases, multivariate analysis indicated that age, fever symptom, prothrombin time, levels of procalcitonin, PaCO_2_ and HbO_2_, and crazy paving patterns in CT manifestations are independent risk factors.

Antibiotics were commonly used in the ICU for ALI patients due to possible bacterial (co)infection. Here, in the COVID-19 cohort, highly effective and broad-spectrum antibiotics were recommended for treatment along with antiviral drugs. At the same time, mechanical ventilation, whether invasive or non-invasive, was applied to support respiration. However, antibiotic treatment did not improve the survival of severe COVID-19 patients, consistent with a recent report (34). Moreover, early administration of antibiotics in severe COVID-19 patients may cause antibiotic resistance in the late stage of treatment (35). Invasive and non-invasive oxygen support by mechanical ventilation did not significantly influence the survival of non-COVID-19 patients. Invasive but not non-invasive mechanical ventilation caused a higher fatality rate in the COVID-19 cohort, as described in other reports (36, 37). This suggested that intubation could further damage lung function and aggravate the condition of critically ill COVID-19 patients, whereas simpler, non-invasive respiratory support should be prioritized. Even with limited understanding of COVID-19 during the beginning of pandemic, antiviral treatments (α-interferon, ribavirin, arbidol, and oseltamivir) applied in our COVID-19 cohort enabled efficacious clearance of SARS-CoV-2 and improved patient prognosis. Validated treatment plans against COVID-19 extend to remdesivir, chloroquine, corticosteroids, convalescent plasma, and monoclonal antibodies (38).

This study has several limitations. First, the sample size is small, which leads to a high variability, causing bias. In this retrospective study, we collected patient data from three regional hospitals, where a limited number of ALI patients had been admitted. Second, given a large set of variables studied but the relatively small size of the sample, the validity of multivariate analysis may be weakened, although it did yield useful information. Third, most blood parameters were not continuously monitored or recorded in the ICU settings. This may restrict our understanding toward the disease development and so limit our conclusion.

In conclusion, our study highlights the distinction in ALI characteristics between severe COVID-19 and non-COVID-19 patients and demonstrated the efficacy of our current therapeutic regimen in the ICU scenario through improved survival of critically ill ALI patients. This work will enhance our understanding of this life-threatening illness and help develop refined treatment regimens leading to better outcomes.

## Data Availability Statement

The data analyzed in this study is subject to the following licenses/restrictions: Data available on request due to privacy/ethical restrictions. Requests to access these datasets should be directed to Zhimin Tao, jsutao@ujs.edu.cn.

## Ethics Statement

The studies involving human participants were reviewed and approved by the Affiliated Hospital of Jiangsu University, Jiangxia First People's Hospital, Huangshi Central Hospital. The patients/participants provided their written informed consent to participate in this study.

## Author Contributions

JiaZ and ZT conceived the idea and designed the study. JiaZ, XH, DD, JinZ, LX, ZH, and ZT contributed to the data acquisition, processing, and table preparation. JiaZ, WX, and ZT contributed to the manuscript writing. JiaZ, XH, and ZT contributed to the statistical analysis. All authors reviewed and approved the manuscript submission.

## Conflict of Interest

The authors declare that the research was conducted in the absence of any commercial or financial relationships that could be construed as a potential conflict of interest.

## Publisher's Note

All claims expressed in this article are solely those of the authors and do not necessarily represent those of their affiliated organizations, or those of the publisher, the editors and the reviewers. Any product that may be evaluated in this article, or claim that may be made by its manufacturer, is not guaranteed or endorsed by the publisher.
