# Supplementary material for: Comparative Study of Acute Lung Injury in COVID-19 and Non-COVID-19 Patients
Source: Front Med (Lausanne). 2021 Aug 16;8:666629. doi: 10.3389/fmed.2021.666629 (PMC8415545; doi:10.3389/fmed.2021.666629)
Supplement: Supplementary file 1 [file Table_1.DOCX]

**Supplementary Information**

**for**

**Comparative study of acute lung injury in COVID-19 and non-COVID-19 patients**

Jianguo Zhang^1,2^, Xing Huang^3^, Daoyin Ding^4^, Jinhui Zhang^2^, Liusheng Xu^1^, Zhenkui Hu^2^, Wenrong Xu^1^, Zhimin Tao^1,*^

^1^Jiangsu Province Key Laboratory of Medical Science and Laboratory Medicine, School of Medicine, Jiangsu University, Zhenjiang, Jiangsu 212013, China

^2^Department of Critical Care Medicine, The Affiliated Hospital, Jiangsu University, Zhenjiang, Jiangsu 212001, China

^3^Center for Evidence-Based and Translational Medicine, and Department of Urology, Zhongnan Hospital of Wuhan University, Wuhan 430071, China

^4^Department of Critical Care Medicine, The First People’s Hospital of Jiangxia District, Wuhan, Hubei 430200, China

^*^ Correspondences should be addressed to:

Zhimin Tao: [jsutao@ujs.edu.cn](mailto:jsutao@ujs.edu.cn)

***Study design***

130 ALI patients admitted to the ICU in the Affiliated Hospital of Jiangsu University (TAHJU) in Zhenjiang, Jiangsu Province, China, from January 2017 to October 2019, were selected as non-COVID-19 cohort. Patient consents were acquired, and the study was approved by the Medical Ethics Committee of TAHJU. In parallel, 90 severe patients in the COVID-19 cohort were admitted by the First People’s Hospital of Jiangxia District (TFPHJD) at Wuhan and Huangshi Central Hospital (HCH) at Huangshi city, both in the Hubei Province, China, during January 15 to April 15, 2020. Patient information remains anonymous, written consent was waived, and the study was individually approved by Ethics Commissions of TFPHJD and HCH. Acute respiratory distress syndrome (ARDS) was defined when positive end expiratory pressure (PEEP) or continuous positive airway pressure (CPAP) was greater than 5 cmH_2_O and PaO_2_/FiO_2_ less than 300 mmHg, following a classic Berlin Definition (Force et al., 2012). ALI/ARDS management was following the published formal guidelines (Chinese Society of Critical Care and Chinese Medical, 2006;Griffiths et al., 2019;Papazian et al., 2019). All data were gathered before and after ICU stays of patients for comparison.

***Procedure***

All COVID-19 patients were received at TFPHJD and HCH and diagnosed by following a standard procedure, and all ICU patients were admitted by following the published criteria(Li, 2020). For patient who had clinical symptoms, such as fever, cough, and radiological abnormality, throat swab samples were gathered for SARS-CoV-2 RNA detection by gene sequencing or real-time RT-PCR, a process with details as previously reported(Huang et al., 2020). For the severe COVID-19 patients in the ICU, they typically developed hypoxemia, dyspnea, and even respiratory failure requiring respiratory support or invasive mechanical ventilation. They were receiving low molecular weight heparin (LMWH, 4-6 kDa) (administered 5000 IU per day via subcutaneous injection unless an increased risk of bleeding was assessed), antibiotic treatment, antiviral drugs, and respiration-assisted ventilation with sedatives (propofol or dexmedetomidine), etc. Blood cell analysis was detected by automated hematology analyzer (SYSMEX 800i, Japan), and the biochemical indicator was analyzed (Toshiba TAB2000, Japan; Roche Cobas 6000 Analyzer, Switzerland). For treated patients whose clinical symptoms disappeared together with improved CT scans, they would be considered cured, followed by discharge from the hospital, only after their nucleic acid tests returned with two continuously negative results on every other day.

130 non-COVID-19 (non-viral) ALI patients were selected who had been treated at TAHJU. All patients following the admission into the ICU were given mechanical ventilation, and the tidal volume was measured to 4-6 mL/kg based on ideal body weight (for mild ARDS patients, 6-8 mL/kg based on ideal body weight was applied per necessity) to maintain the airway platform pressure <30cm H_2_O. A continuous positive end-expiratory pressure support was applied, while 8.0cm H_2_O support could be first performed depending on the respiratory status of the patient, and later adjusted according to the oxygenation state of the patient. All patients were given active treatment of their primary disease and strict control of blood sugar. For infection treatment, imipenem/cilastatin, sulbactam/cefoperazone sodium, or/and piperacillin/sulbactam sodium were given via injection based on the body weight of the patient. ICU patients were also receiving LMWH (4-6 kDa) as administered 5000 IU per day via subcutaneous injection, unless an increased risk of bleeding was assessed. For improvement of wet cough, ambroxol hydrochloride was intravenously injected to dilute and expel the phlegm. Upon mechanical ventilation, midazolam, propofol or dexmedetomidine was given via intravenous pumping, and patients were kept in light sedation.

[***Statistical***](C:/Users/nowhere%20man/AppData/Local/youdao/dict/Application/8.5.3.0/resultui/html/index.html#/javascript:;) [***analysis***](C:/Users/nowhere%20man/AppData/Local/youdao/dict/Application/8.5.3.0/resultui/html/index.html#/javascript:;)

The categorical variables were described as frequency rates and percentages, and continuous variables were applied to describe the median and quartile range (IQR) values. Comparison of continuous variables between two groups was analyzed with Mann-Whitney test. Repeated measurements (non-normal distribution) were used following a generalized linear mixed model. χ^2^ test was used to compare the proportion of categorical variables, and the Fisher exact test was employed when data was limited. All statistical analyses were performed using GraphPad Prism 5.0 software (GraphPad Software, Inc., San Diego, CA). A two-sided α of less than 0.05 was considered statistically significant unless otherwise specified.


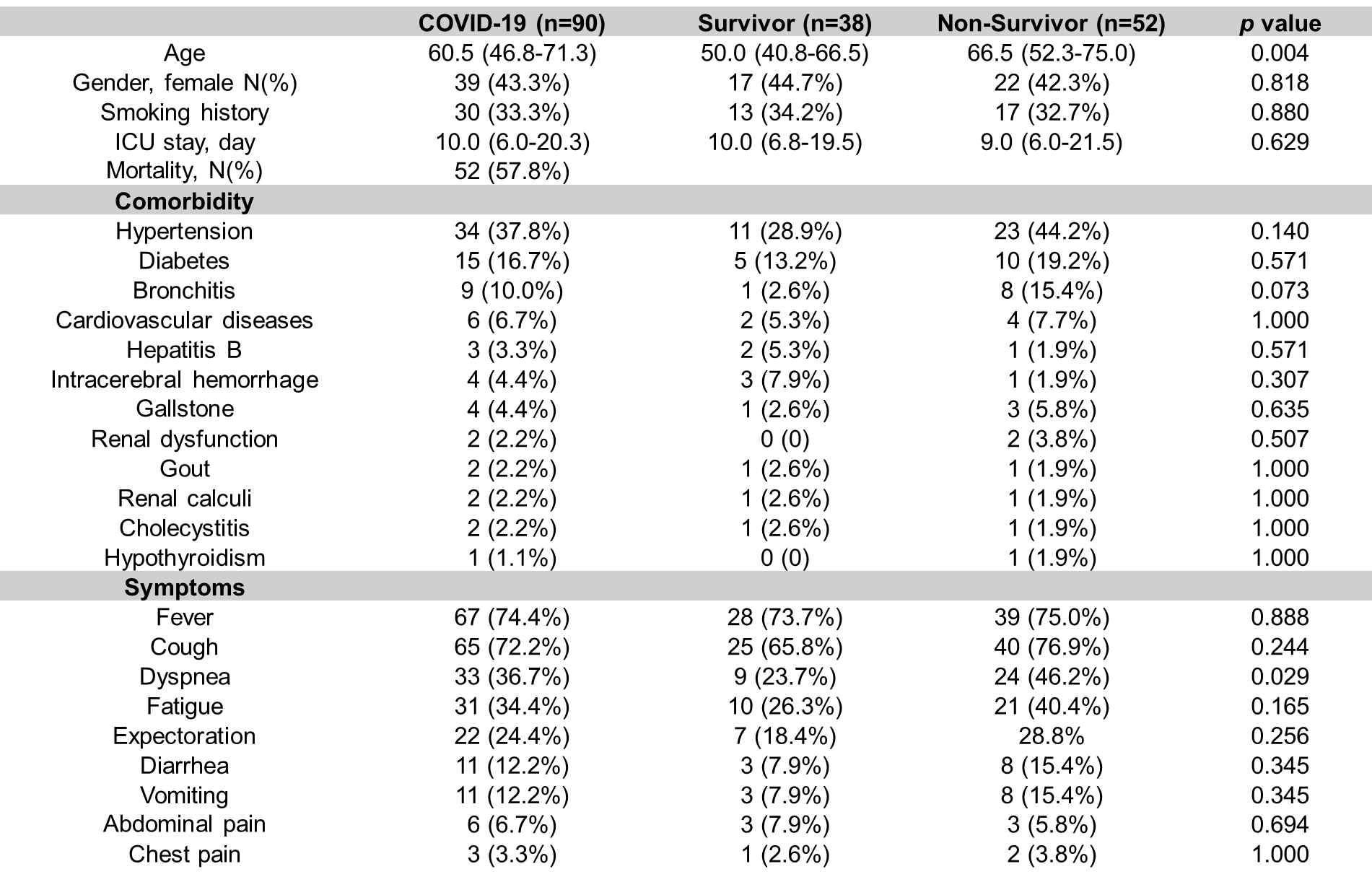
Table S1. Demographic information and medical history of ALI patients in the COVID-19 cohort.


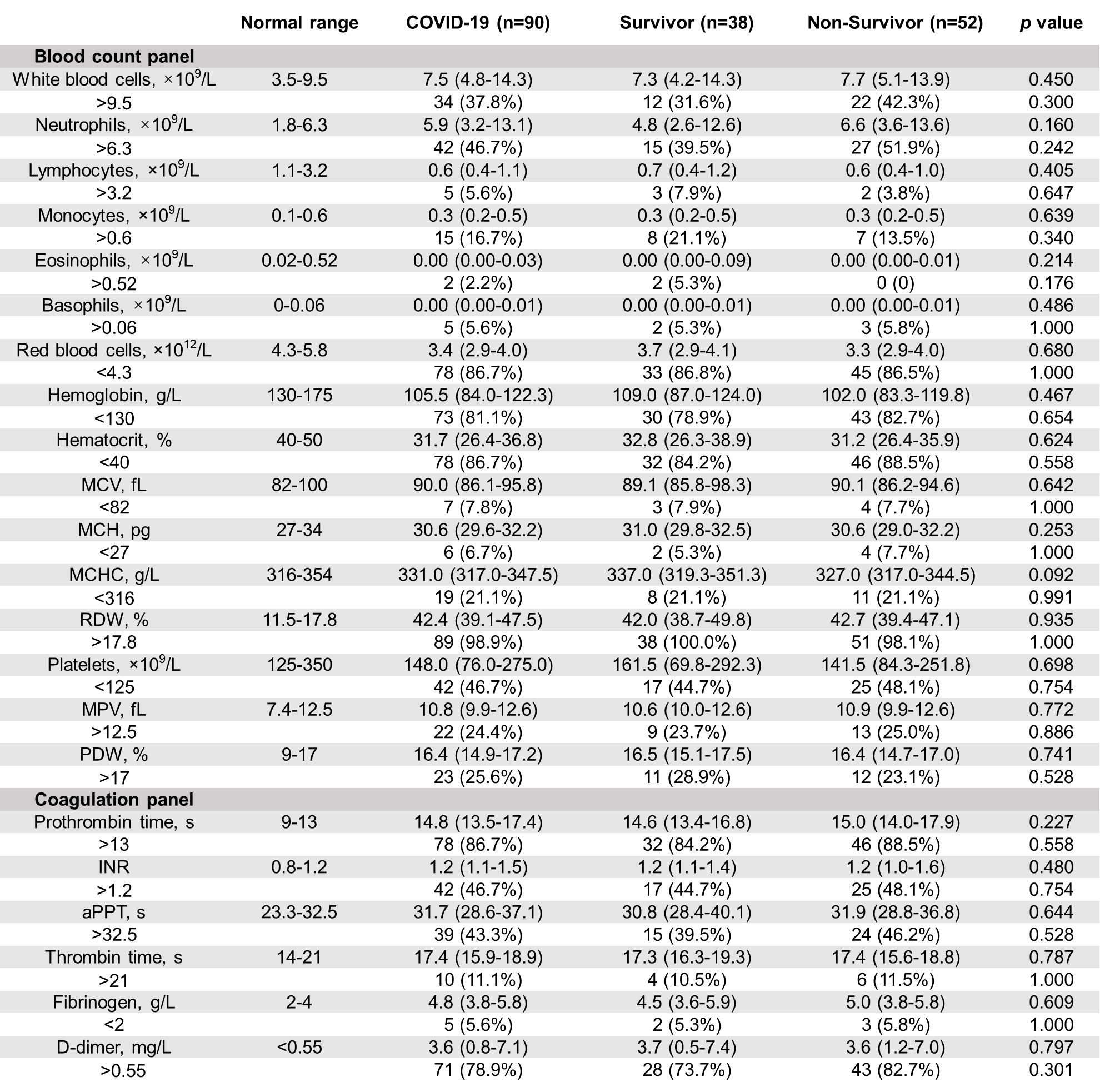
Table S2. Laboratory testing results of ALI patients in the COVID-19 cohort.


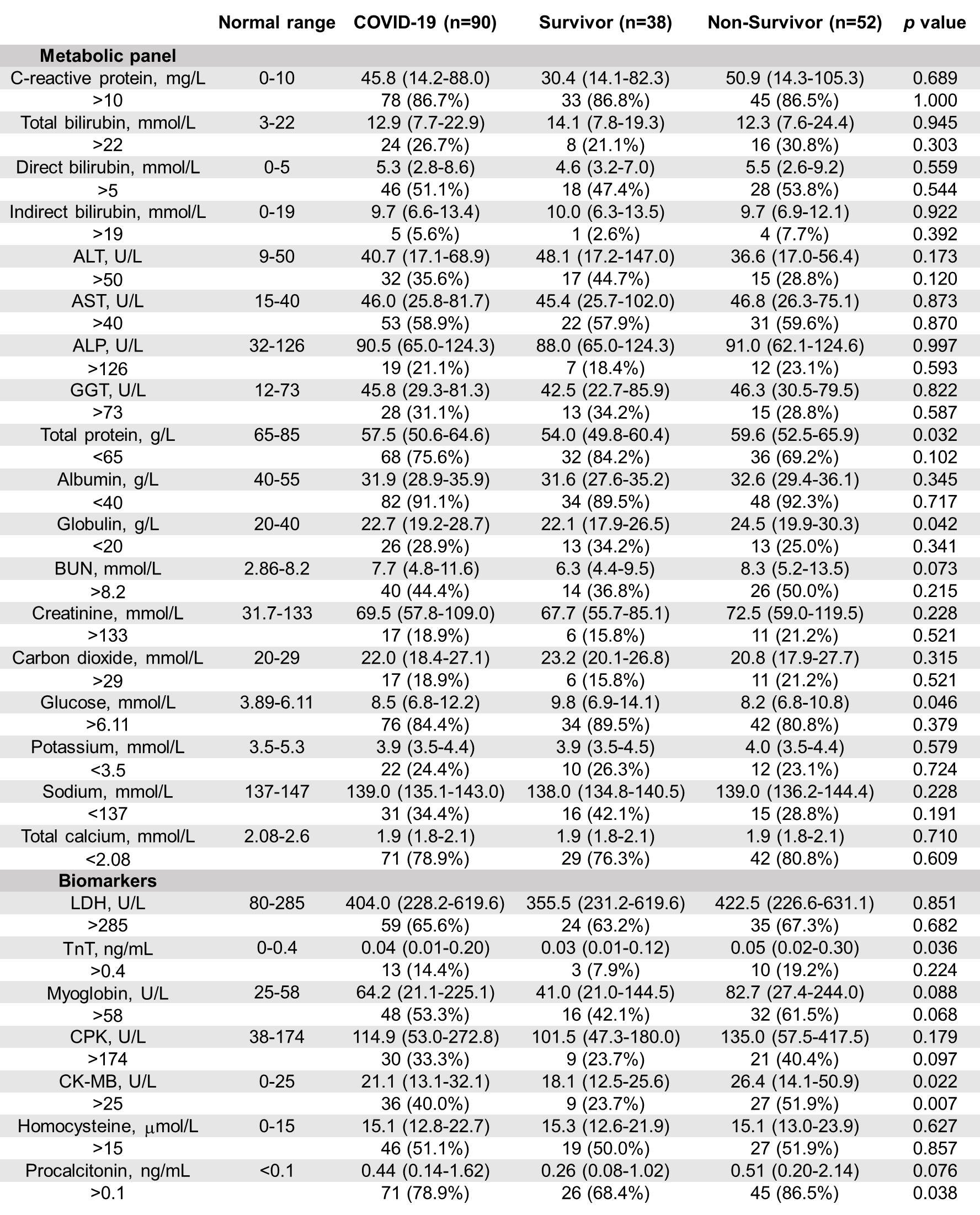


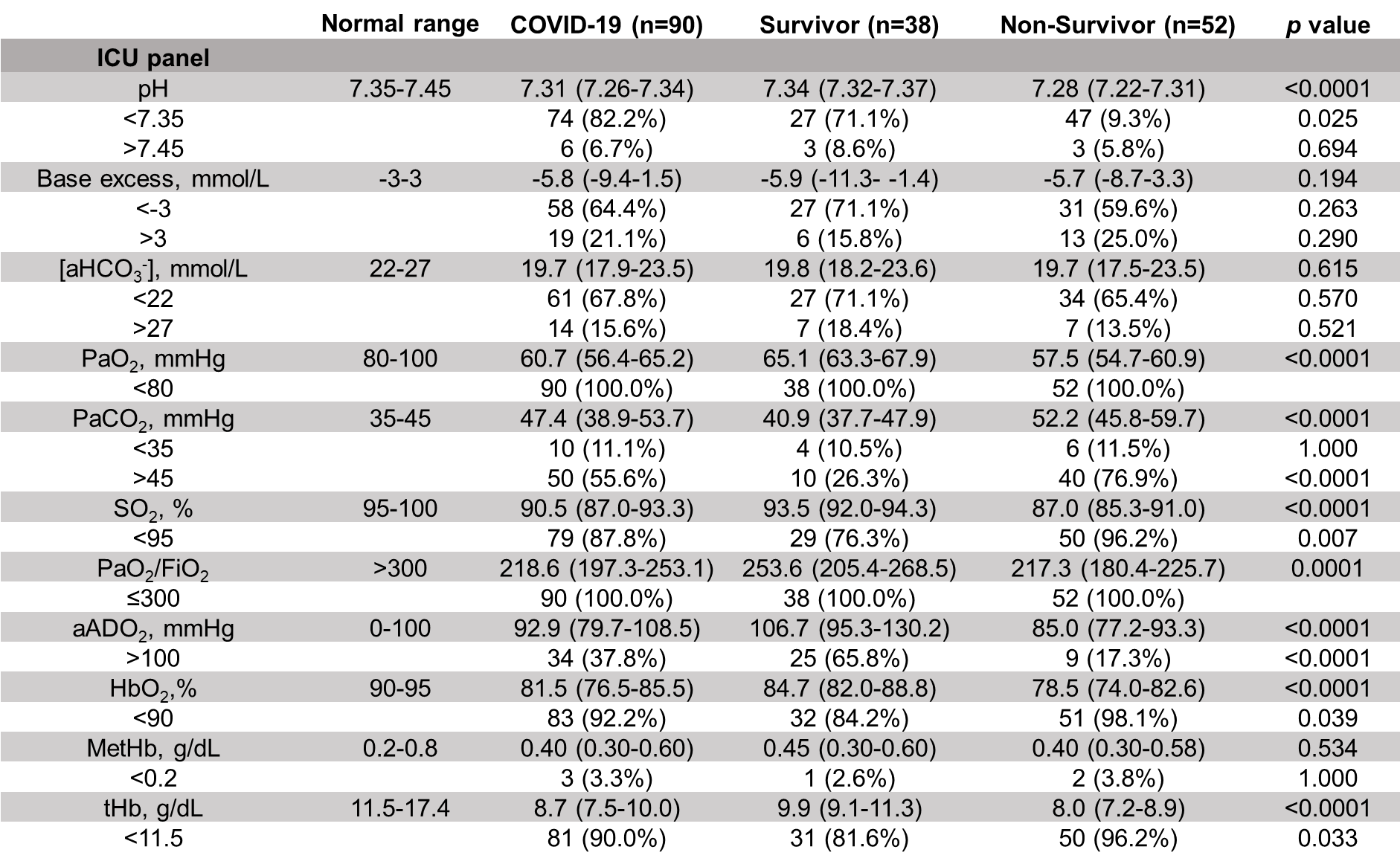
Table S3. Arterial blood gas profiles for ALI patients in the COVID-19 cohort.


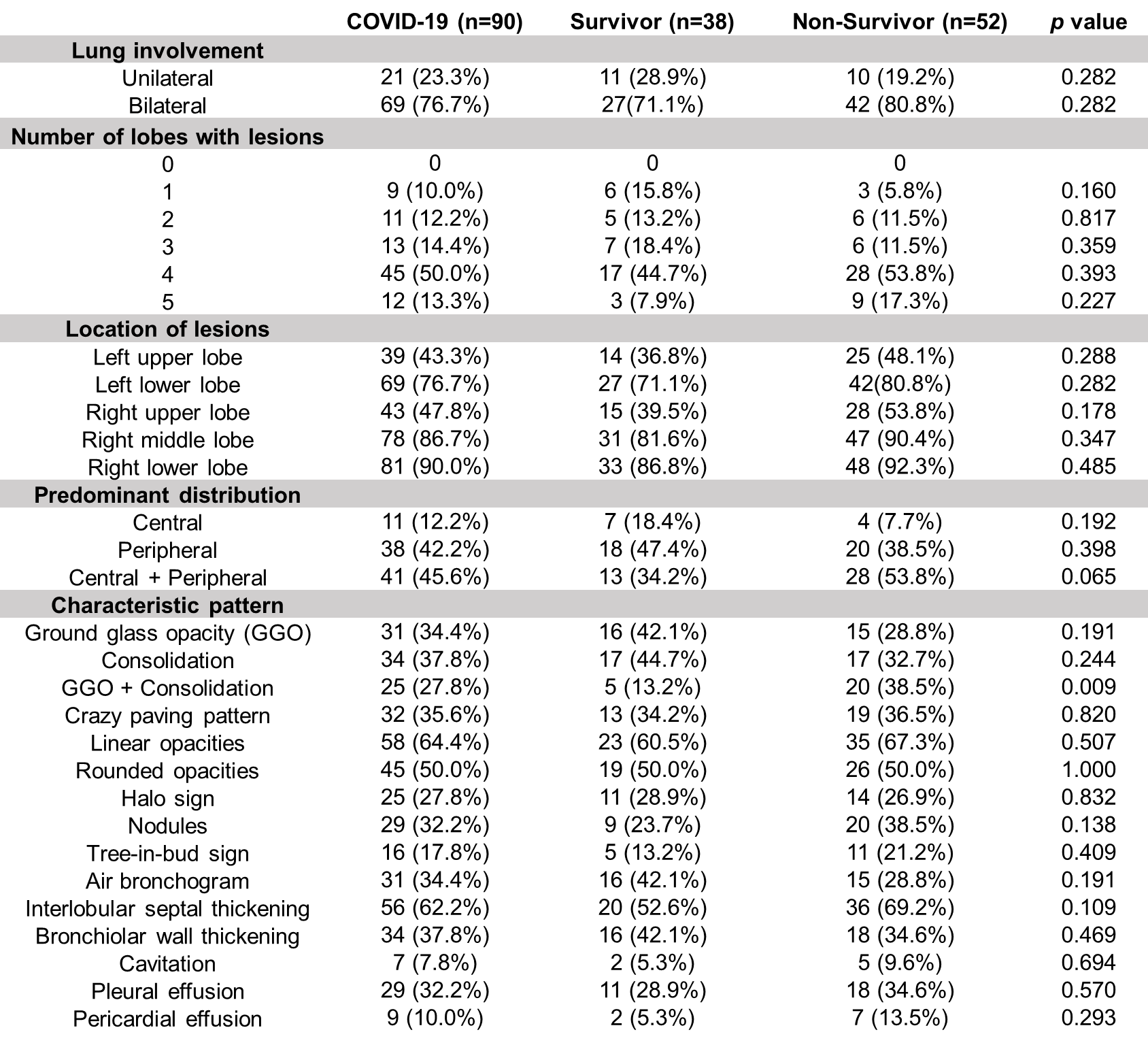
Table S4. Radiological findings of ALI patients in the COVID-19 cohort.


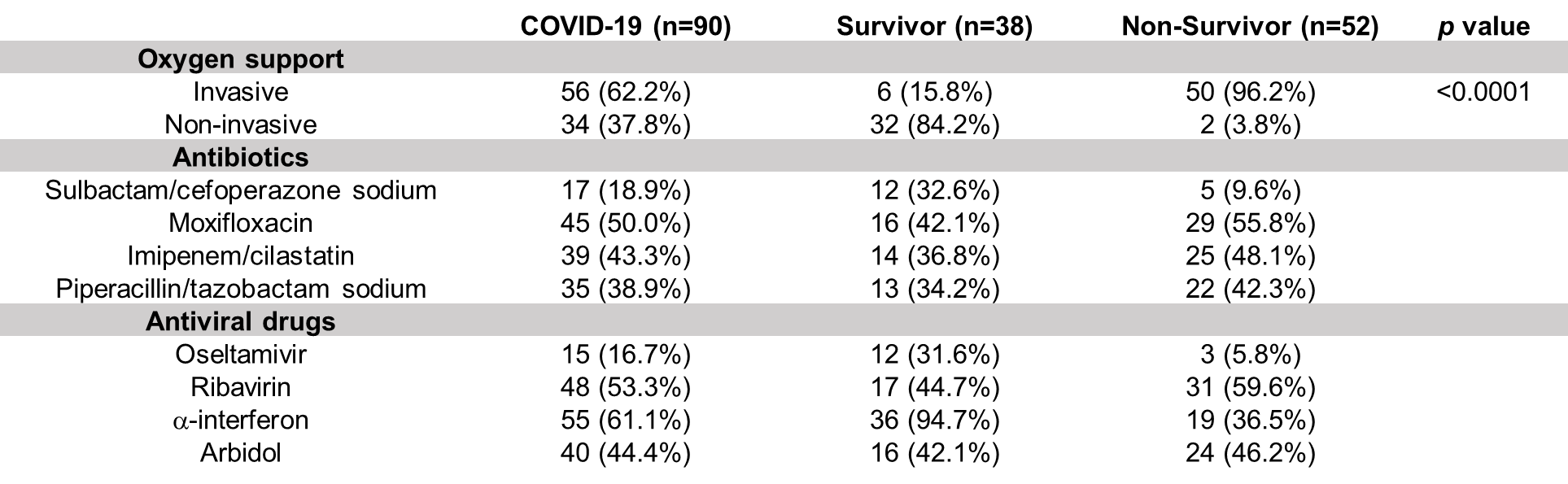
Table S5. Treatment of ALI patients in the COVID-19 cohort.


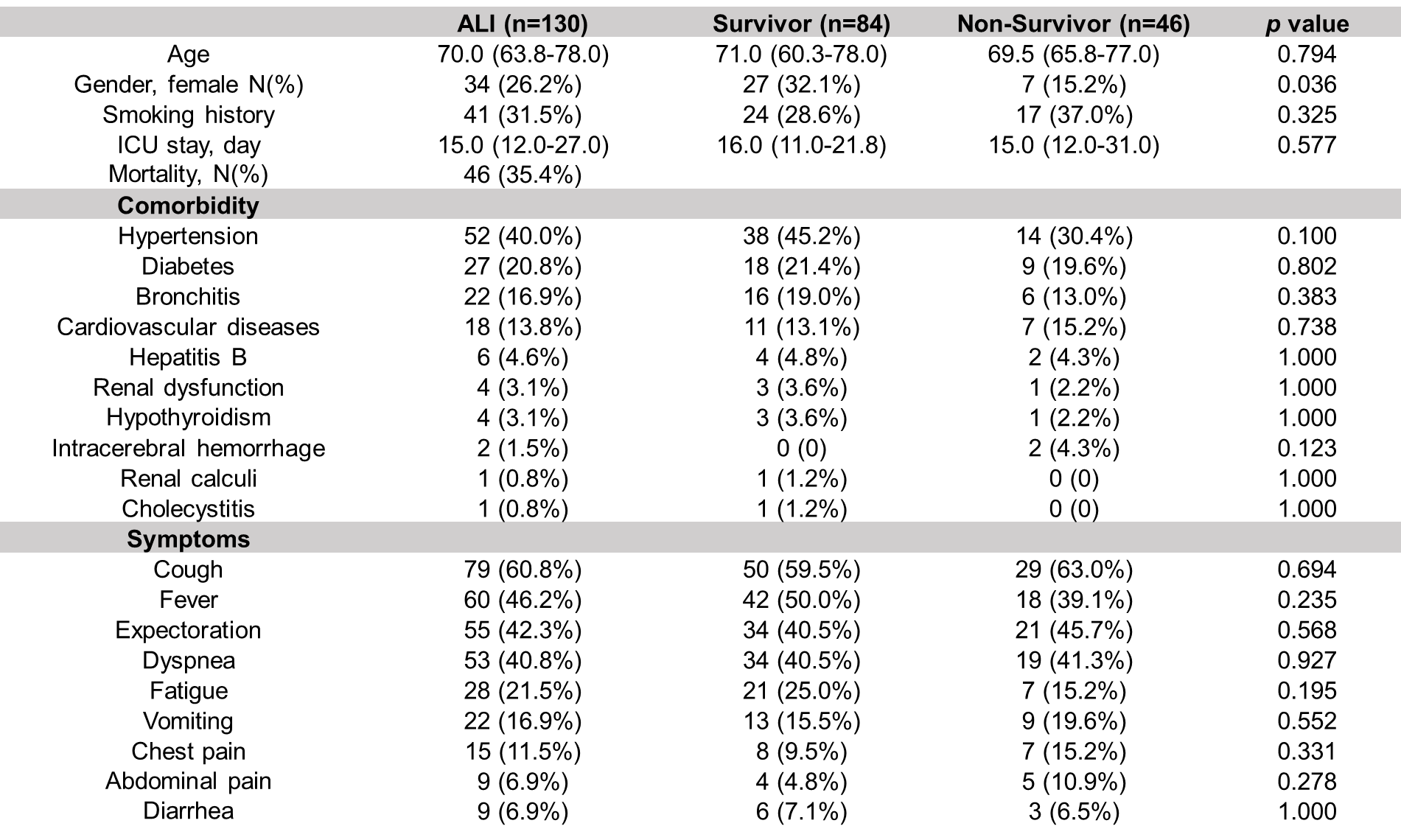
Table S6. Demographic information and medical history of ALI patients in the non-COVID-19 cohort.


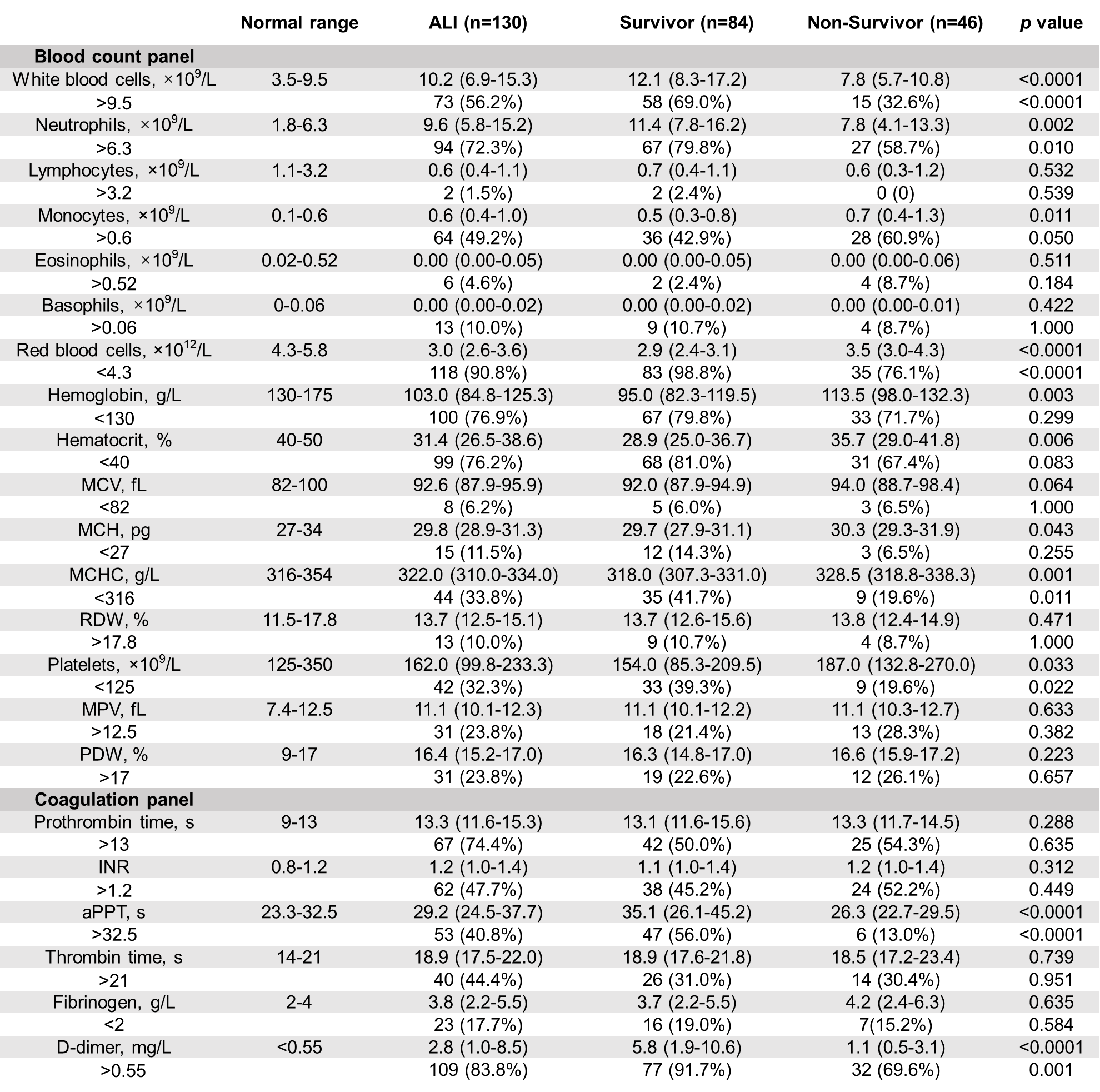
Table S7. Laboratory testing results of ALI patients in the non-COVID-19 cohort.


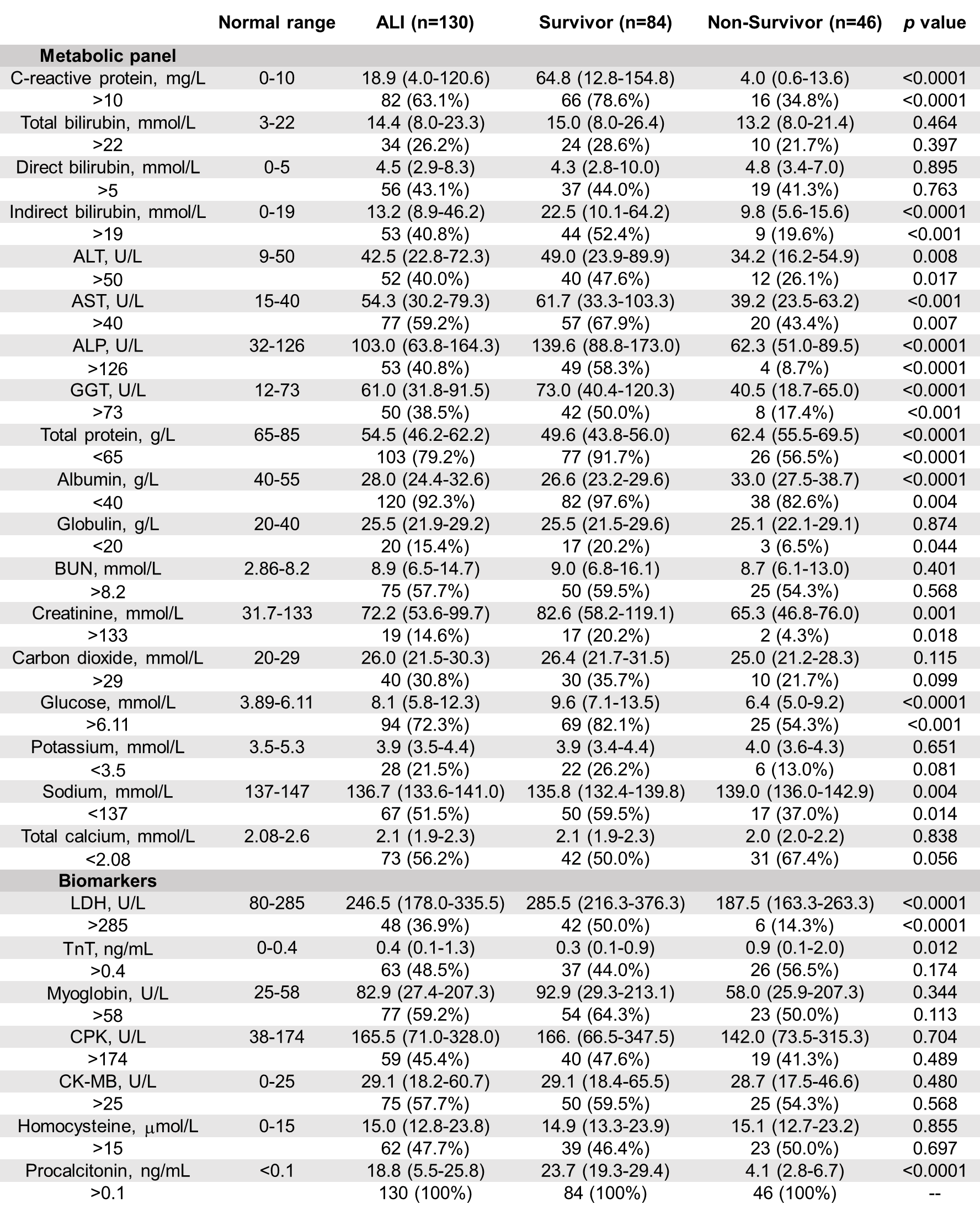


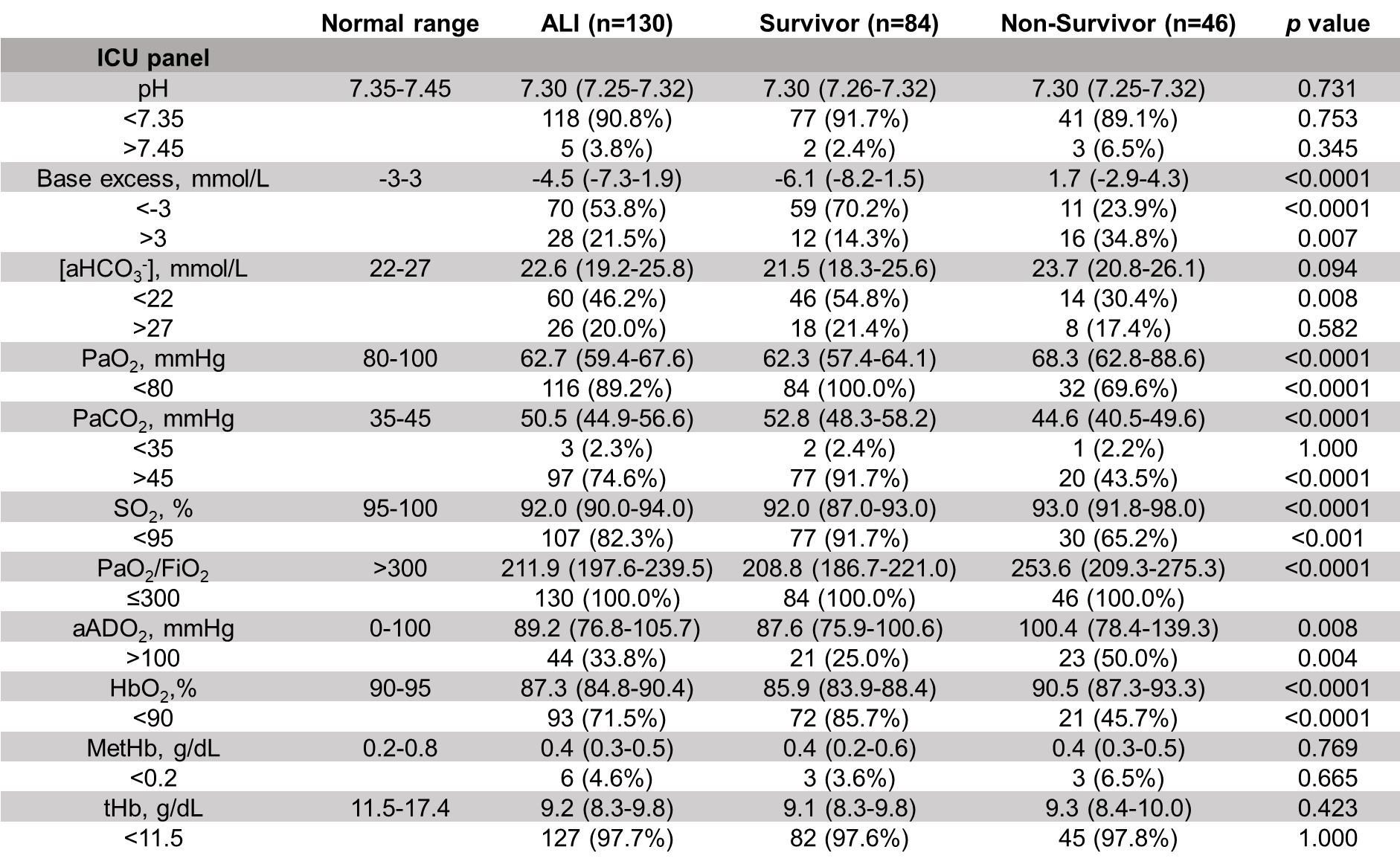
Table S8. Arterial blood gas profiles for ALI patients in the non-COVID-19 cohort.


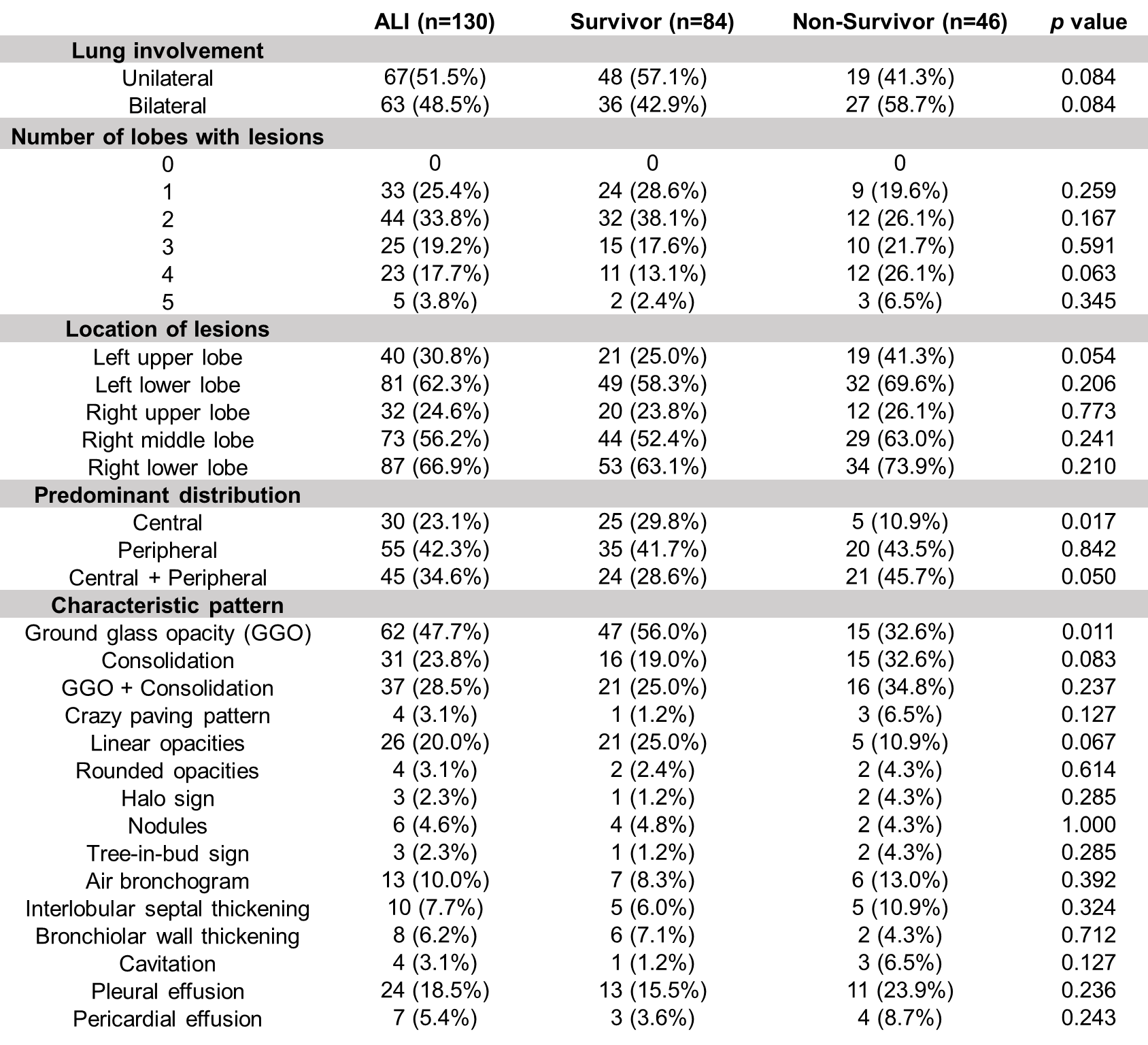
Table S9. Radiological findings of ALI patients in the non-COVID-19 cohort.


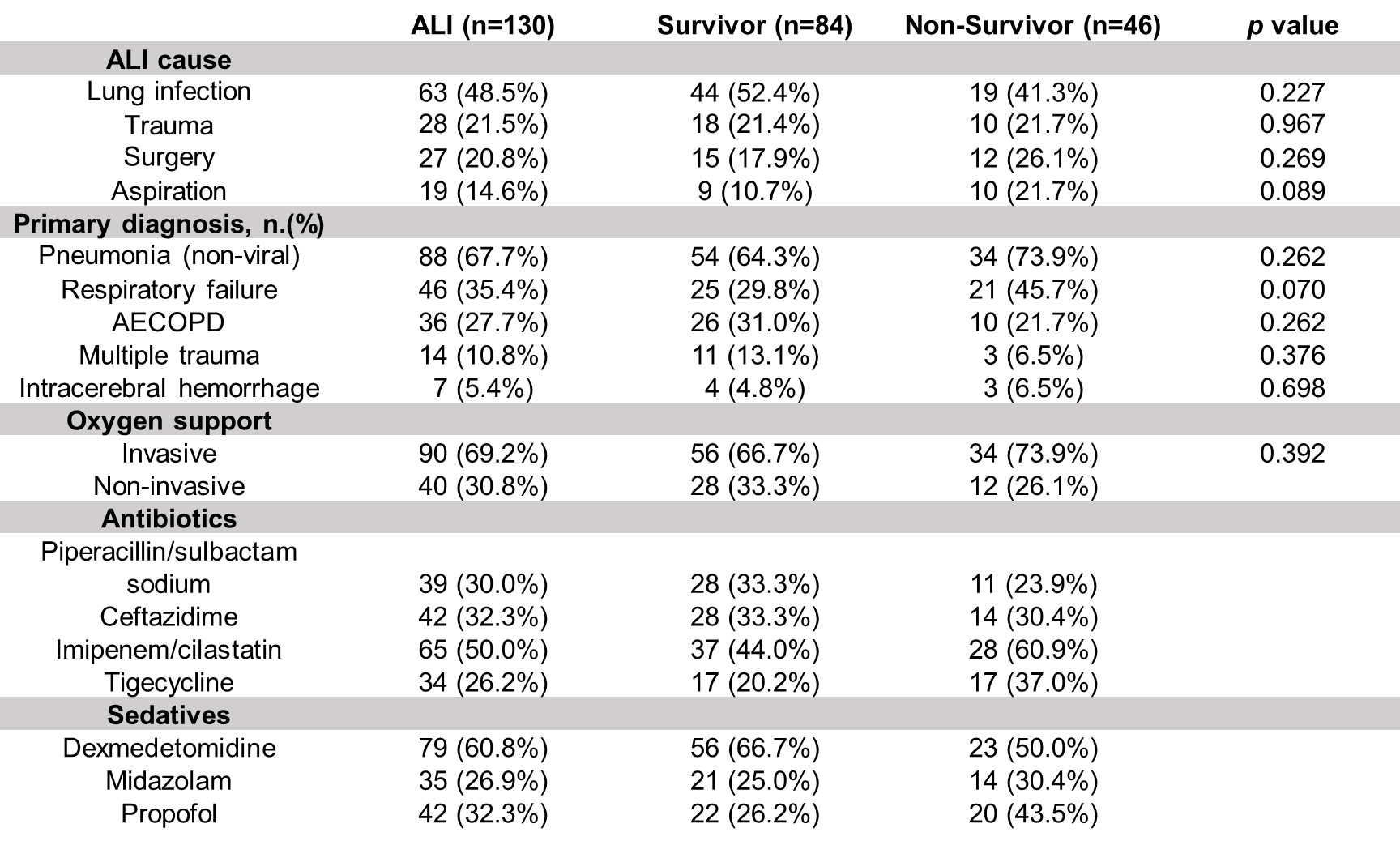
Table S10. Cause of ALI, primary diagnosis, and treatment of ALI patients in the non-COVID-19 cohort.

Table S11. Laboratory testing results of ALI survivors in the COVID-19 cohort
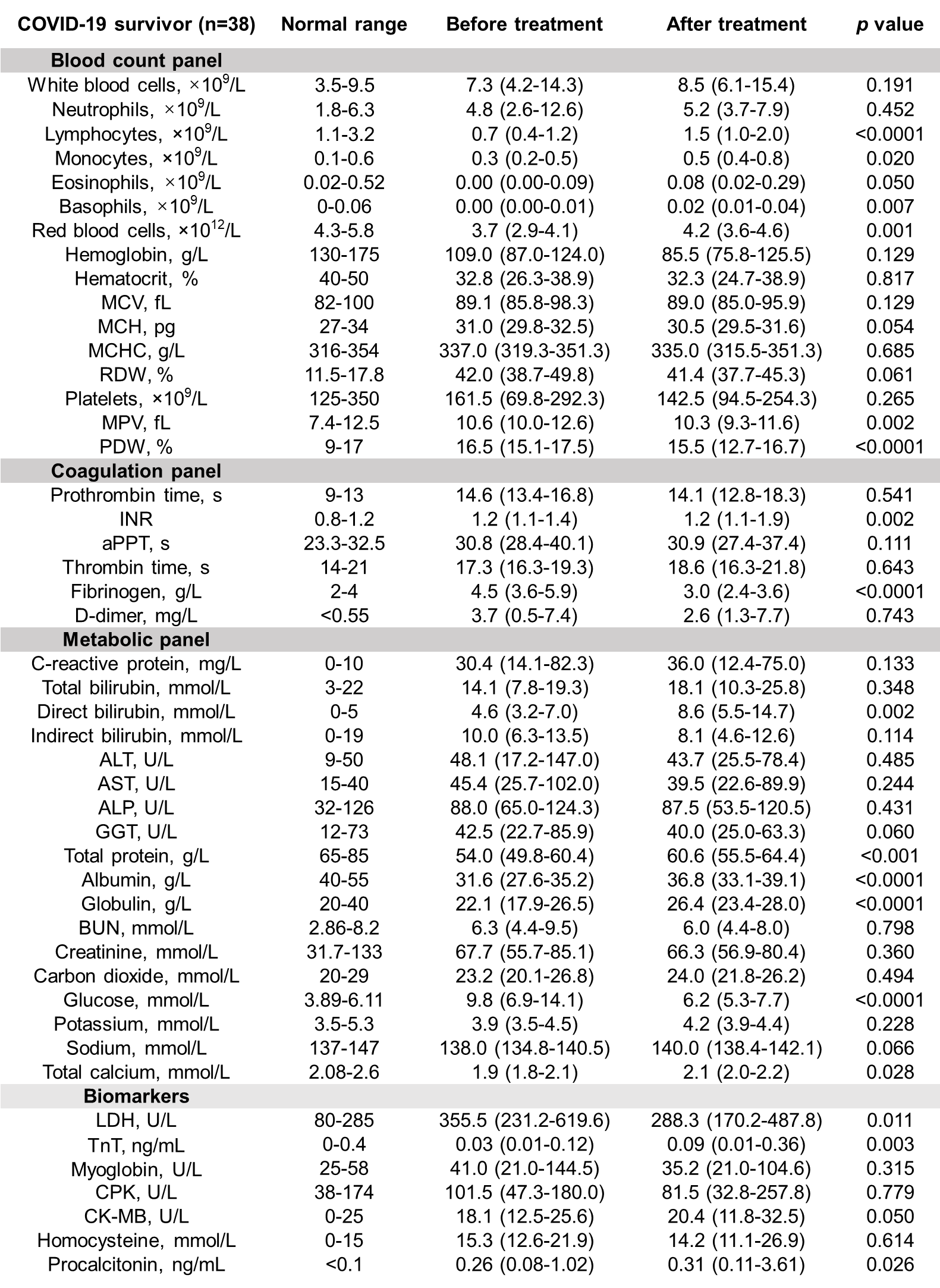
before and after treatment.


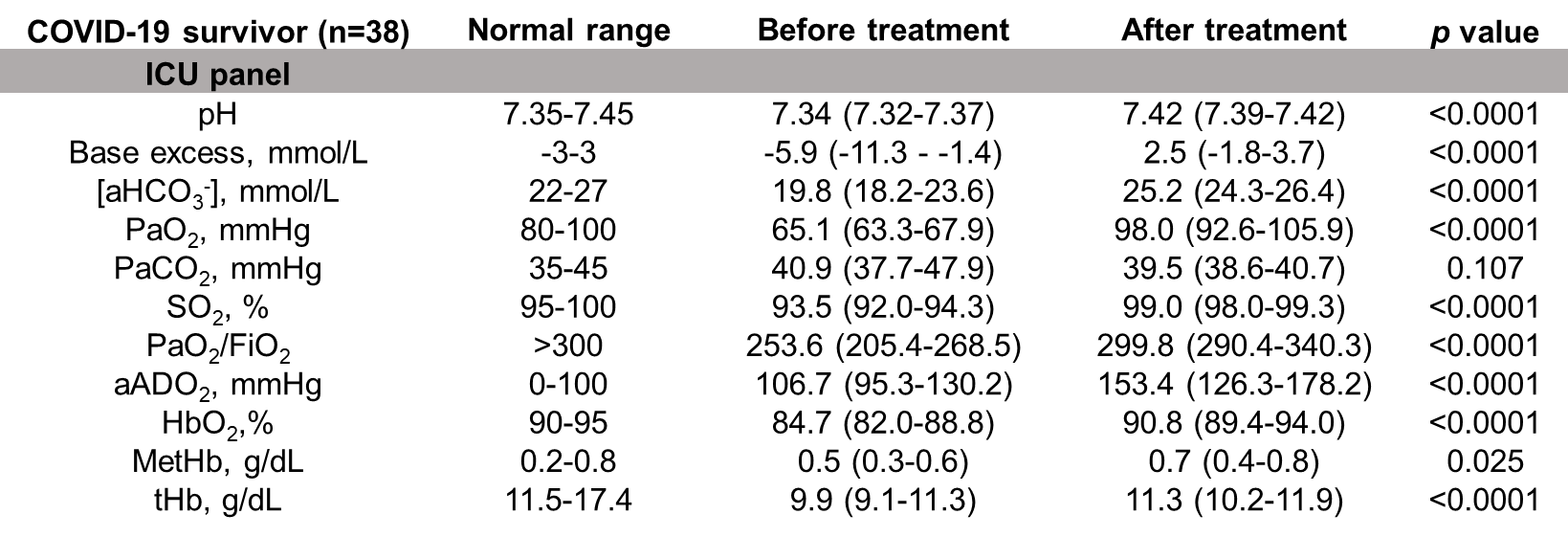
Table S12. Arterial blood gas profiles for ALI survivors in the COVID-19 cohort before and after treatment.

Table S13. Radiological findings of ALI survivors in the COVID-19 cohort before and after treatment.


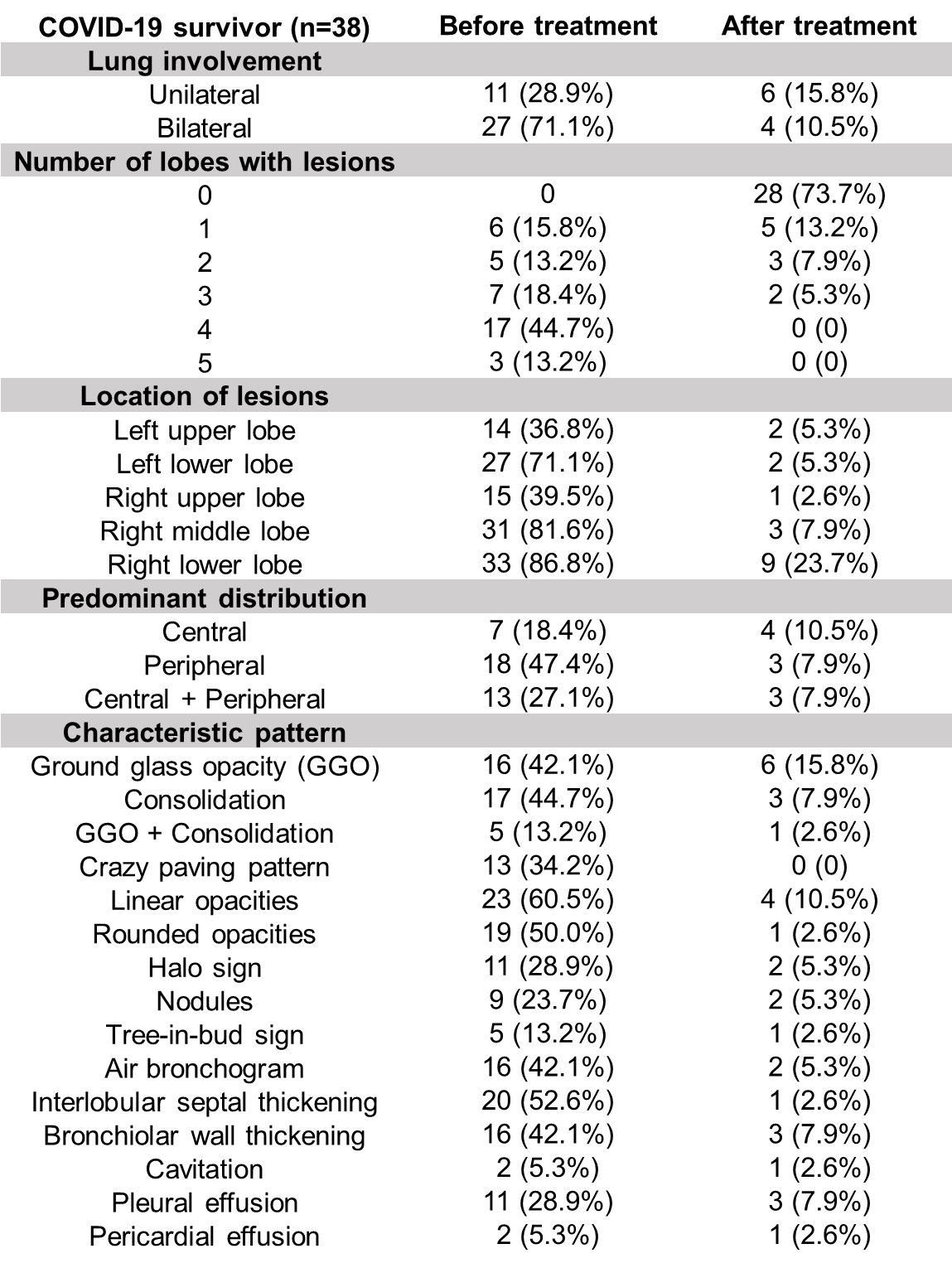


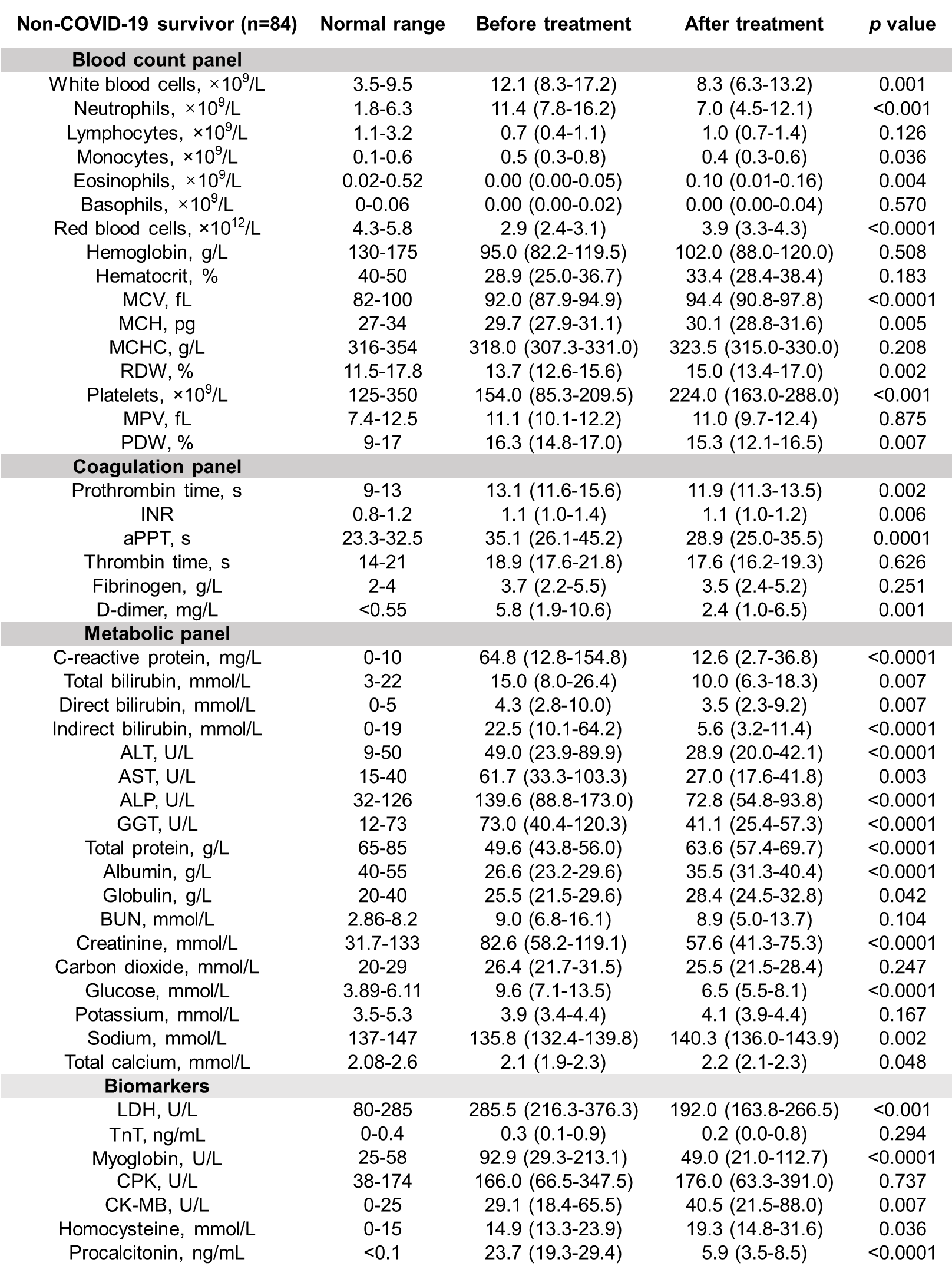
Table S14. Laboratory testing results of ALI survivors in the non-COVID-19 cohort before and after treatment.


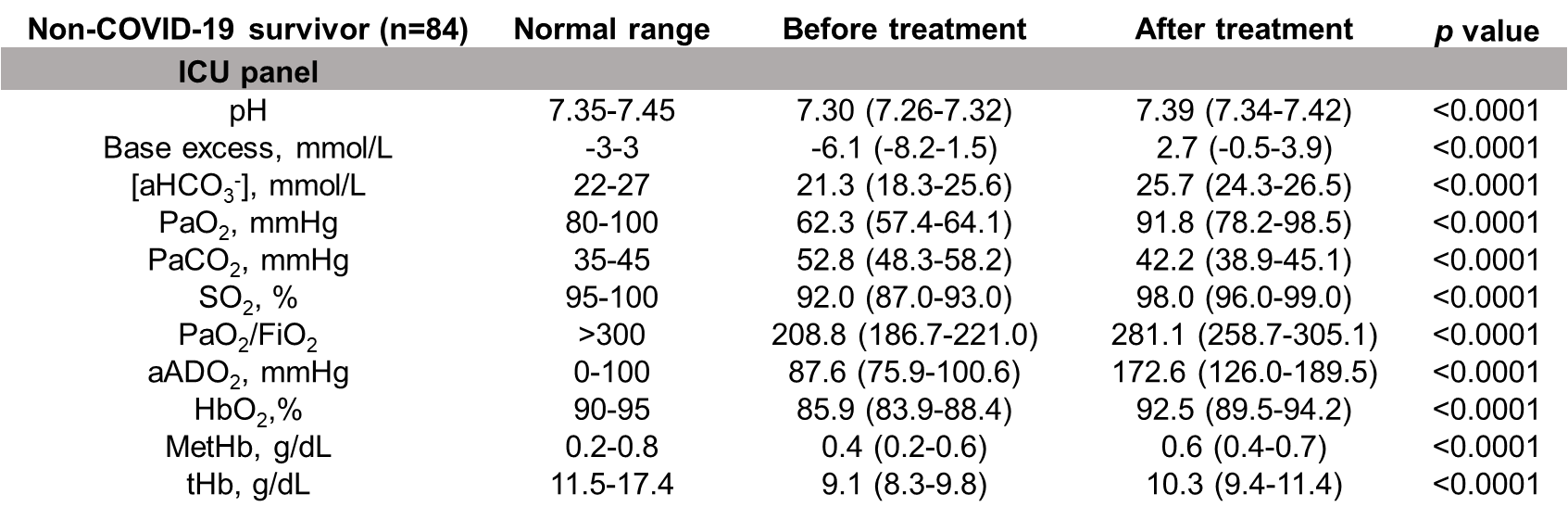
Table S15. Arterial blood gas profiles for ALI survivors in the non-COVID-19 cohort before and after treatment.

Table S16. Radiological findings of ALI survivors in the non-COVID-19 cohort before and after treatment.


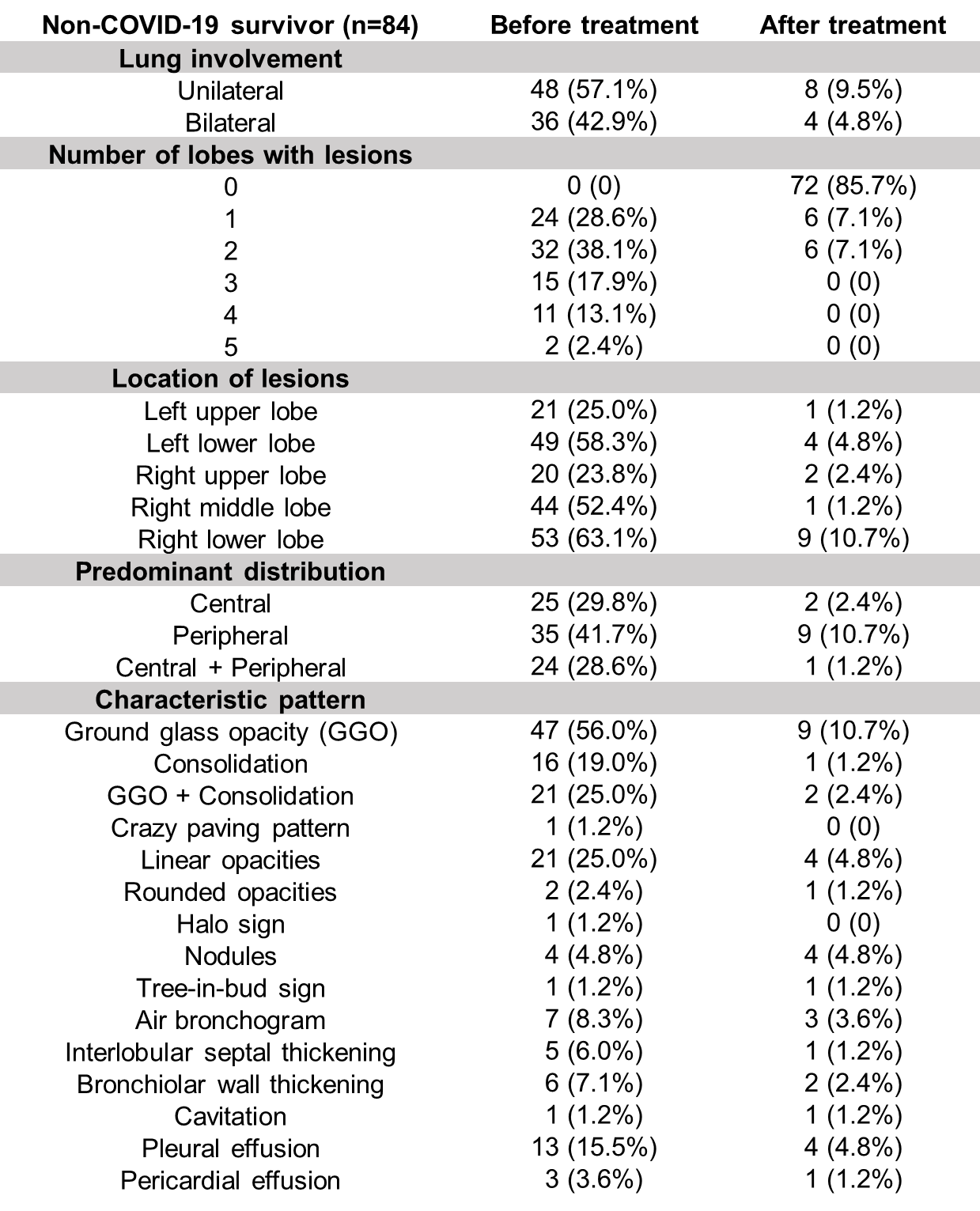


**References**

Chinese Society of Critical Care, M., and Chinese Medical, A. (2006). [Guidelines for management of acute lung injury/acute respiratory distress syndrome: an evidence-based update by the Chinese Society of Critical Care Medicine (2006)]. *Zhongguo Wei Zhong Bing Ji Jiu Yi Xue* 18**,** 706-710.

Force, A.D.T., Ranieri, V.M., Rubenfeld, G.D., Thompson, B.T., Ferguson, N.D., Caldwell, E., Fan, E., Camporota, L., and Slutsky, A.S. (2012). Acute respiratory distress syndrome: the Berlin Definition. *JAMA* 307**,** 2526-2533.

Griffiths, M.J.D., Mcauley, D.F., Perkins, G.D., Barrett, N., Blackwood, B., Boyle, A., Chee, N., Connolly, B., Dark, P., Finney, S., Salam, A., Silversides, J., Tarmey, N., Wise, M.P., and Baudouin, S.V. (2019). Guidelines on the management of acute respiratory distress syndrome. *BMJ Open Respir Res* 6**,** e000420.

Huang, C., Wang, Y., Li, X., Ren, L., Zhao, J., Hu, Y., Zhang, L., Fan, G., Xu, J., Gu, X., Cheng, Z., Yu, T., Xia, J., Wei, Y., Wu, W., Xie, X., Yin, W., Li, H., Liu, M., Xiao, Y., Gao, H., Guo, L., Xie, J., Wang, G., Jiang, R., Gao, Z., Jin, Q., Wang, J., and Cao, B. (2020). Clinical features of patients infected with 2019 novel coronavirus in Wuhan, China. *Lancet* 395**,** 497-506.

Li, T. (2020). Diagnosis and clinical management of severe acute respiratory syndrome Coronavirus 2 (SARS-CoV-2) infection: an operational recommendation of Peking Union Medical College Hospital (V2.0). *Emerg Microbes Infect* 9**,** 582-585.

Papazian, L., Aubron, C., Brochard, L., Chiche, J.D., Combes, A., Dreyfuss, D., Forel, J.M., Guerin, C., Jaber, S., Mekontso-Dessap, A., Mercat, A., Richard, J.C., Roux, D., Vieillard-Baron, A., and Faure, H. (2019). Formal guidelines: management of acute respiratory distress syndrome. *Ann Intensive Care* 9**,** 69.
